# ﻿Description of a new *Kurixalus* species (Rhacophoridae, Anura) and a northwards range extension of the genus

**DOI:** 10.3897/zookeys.1108.81725

**Published:** 2022-06-23

**Authors:** Kevin R. Messenger, Siti N. Othman, Ming-Feng Chuang, Yi Yang, Amaël Borzée

**Affiliations:** 1 Herpetology and Applied Conservation Lab, College of Biology and the Environment, Nanjing Forestry University, 159 Longpan Rd, Nanjing, Jiangsu 210037 China; 2 Laboratory of Animal Behaviour and Conservation, College of Biology and the Environment, Nanjing Forestry University, 159 Longpan Rd, Nanjing, Jiangsu 210037 China; 3 Department of Life Sciences and Research Center for Global Change Biology, National Chung Hsing University, No. 145 Xingda Rd., South Dist., Taichung 40227, Taiwan

**Keywords:** Bush frog, China, East Asia, species description, taxonomy, Rhacophorid

## Abstract

Knowledge of biodiversity before species become extinct is paramount to conservation, especially when the relevant species are far from their expected distribution and, thus, likely overlooked. Here, we describe a new *Kurixalus* species corresponding to a range extension of *Kurixalus* on the Asian mainland, with the closest population in Taiwan. The species diverged from its closest relative during the Late Pliocene to Pleistocene, ca. 3.06 Mya (HPD 95%: 5.82-0.01), based on calibrations with a relaxed clock species tree of unlinked mtDNA 12S rRNA and nuclear DNA *TYR*. The status of the newly-described species is also supported by a divergence in call properties and morphometrics. We named the species described here as *Kurixalusinexpectatus* sp. nov. due to the nature of the discovery, as well as the adjunct distribution of the species relative to its closest congeners. The species was found in Zhejiang Province and it represents a range extension of 663 km for the *Kurixalus* genus.

## ﻿Introduction

The taxonomy of the genus *Kurixalus* Ye, Fei & Dubois in Fei (Fei 1999) is still in flux, with the latest species description in 2021 (Zeng et al. 2021). The taxonomy of the family Rhacophoridae follows the same pattern with numerous taxonomic questions still unresolved (Meegaskumbura et al. 2015; Chan et al. 2020; Nguyen et al. 2020). The genus is found throughout south-central and south-eastern Asia, from as far west as northeast India, to as far south as Indonesia, as far east as the Ryuku Islands of Japan and north to the Himalayas and Taiwan (Frost 2020). The genus is often associated with bamboo forests (Chuang et al. 2019; Nguyen et al. 2020) although given the immense range of the genus, it is also present in several other habitat types (Nguyen et al. 2020). Outside of the Himalayas, in mainland China, its northernmost distribution is Chengdu, Sichuan at 30.36°N (Hou et al. 2021) and is also found in northern Taiwan at 25.3°N (Wu et al. 2016; Frost 2020). However, other genera of the family such as *Gracixalus* spp. are found as far north as Mt. Jinggang in Jiangxi Province (Wang et al. 2018) and *Zhangixalusdennysi* is found as far north as Jiangsu (van Dijk et al. 2004).

Many anurans in Asia have undergone several taxonomic changes in the last decade and continue to undergo massive re-assignments at the generic level, such as: *Adenopleura*, *Bufo*, *Hyla* sensu lato (s.l.), *Megophrys* s.l., *Polypedates* s.l., *Rana* s.l. and *Theloderma* s.l., just to name a few (Li and Wang 2008; Li et al. 2009; Chen et al. 2017). The genera within Rhacophoridae have undergone similar massive and frequent re-assignments. Within the *Kurixalus* complex, other genera that have been scrutinised include *Aquixalus* Delorme, Dubois, Grosjean and Ohler 2005 (Delorme et al. 2005), *Chiromantis*Peters 1854 (Peters 1854), *Gracixalus* Delorme, Dubois, Grosjean and Ohler 2005 (Delorme et al. 2005), *Liuixalus* Li, Che, Bain, Zhao and Zhang 2008 (Li et al. 2008), *Nasutixalus*, *Nyctixalus*, *Philautus* and *Zhangixalus* Li, Jiang, Ren and Jiang 2019 (Jiang et al. 2019). Species within these genera have bounced around from one genus or another. To compound matters, many authors seem to frequently disagree on the specific arrangement of a species within a single genus, such as *Kurixalushainanus* Zhao, Wang & Shi, 2005 (Zhao et al. 2005) being considered a junior synonym of *K.odontotarsus* Ye and Fei 1993 (Ye et al. 1993) by Fei et al. (Fei et al. 2010) or a junior synonym of *K.bisacculus*Taylor 1962 (Taylor 1962; Yu et al. 2010). Such doubt in this taxonomic group has made it obvious that more careful inspection of this complex is needed. It is important to note that *Kurixalus* s.l. is now assigned to three independent clades with parapatric distributions (Nguyen et al. 2020): a southern clade on Sundaland assigned to *Zhangixalusappendiculatus* and *K.chaseni*, a continental Asia-restricted clade corresponding to *Aquixalus* (Delorme et al. 2005) and *Kurixalus* s. str. Boettger, 1895 (Boettger 1895) on Taiwan and Ryukus Islands, with some species on southern southeast Asia (Yu et al. 2017b; Lv et al. 2018).

Species in the genus *Kurixalus* are morphologically similar and species identification is difficult (Nguyen et al. 2020). Numerous narrow ranging clades are distributed in South East Asia, with numerous likely undescribed species (Yu et al. 2017a; Lv et al. 2018) and integrated studies that include genetics, call properties, morphology and ecological preferences are required to differentiate the clades (Gonzalez et al. 2014; Yu et al. 2017a; Yu et al. 2018). For instance, only broad sampling was able to highlight the segregated species status between *K.chaseni* from peninsular Malaysia and Borneo and *Z.appendiculatus* from the Philippines (Matsui et al. 2018). The situation is similar in China, where numerous micro-endemics are present and numerous species still need to be described (Yu et al. 2017a).

During herpetological surveys in April and July 2018, we found an unknown frog that could be allocated to family Rhacophoridae, subfamily Rhacophorinae, genus *Kurixalus*Ye et al. (1993), based on serrated dermal fringes of the upper side of the upper arm and tarsus, protruding nostrils, pointed snout and an indistinct tympanum, but could not be assigned to any specific species. Here, we report on a new species of *Kurixalus* from central-eastern China that is highly disjunct (663 km) from the next closest known population of *Kurixalus*. The population represents the northernmost latitude of the genus known to date.

## ﻿Materials and methods

### ﻿Sampling

**Figure 1.**
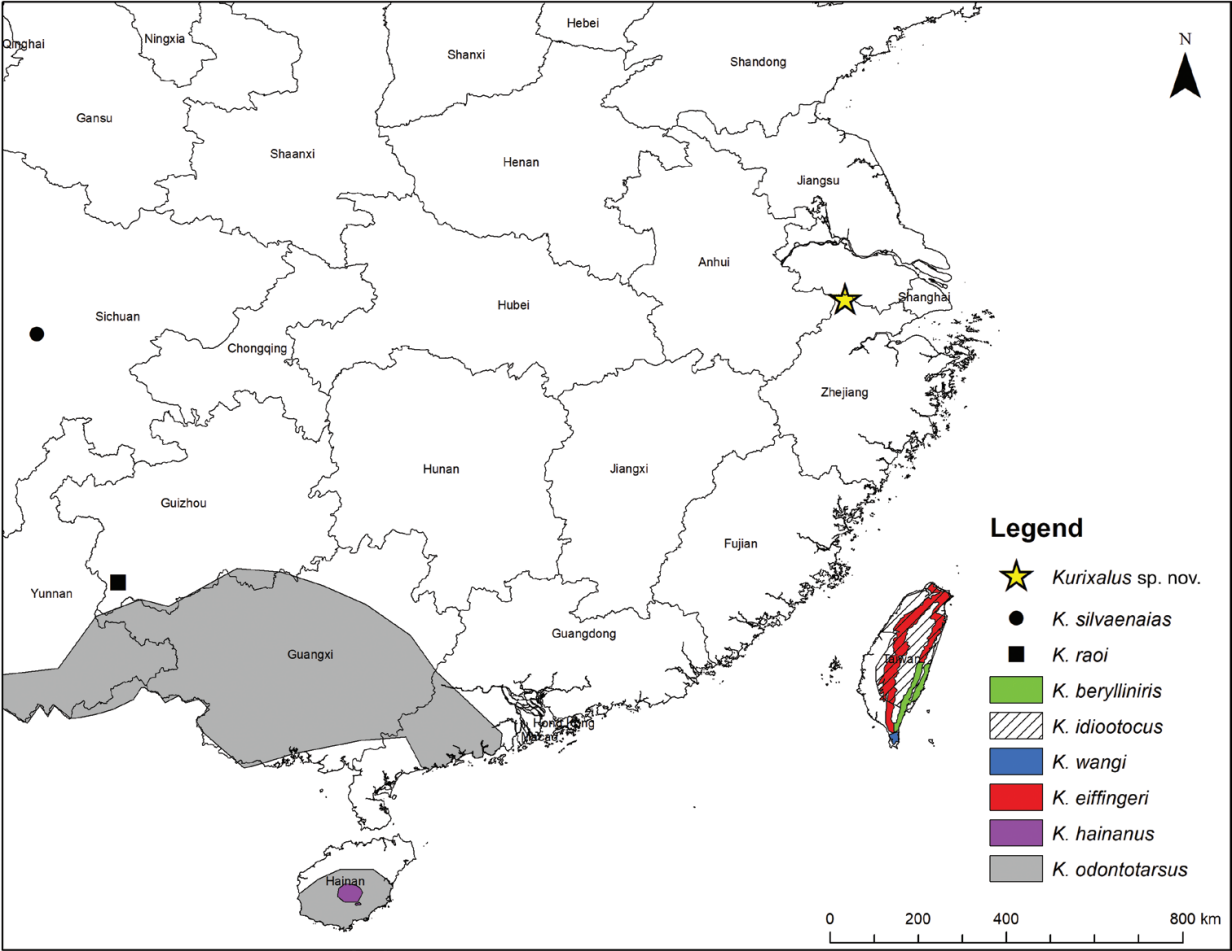
Map of sampling site and *Kurixalus* species. The sample for *Kurixalus* sp. nov. were collected in April and July 2018 in north-western Zhejiang Province, People’s Republic of China. Map generated in ArcMap 10.4.

**Figure 2.**
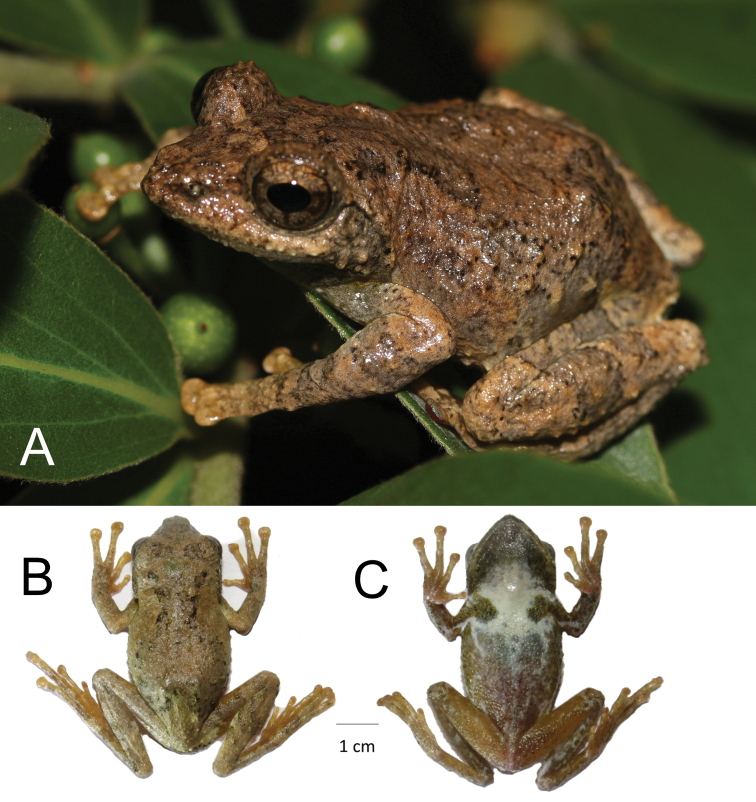
**A***Kurixalus* sp. nov. specimen (HM 323117) *in-situ* from 26 April 2018 and **B** dorsal and **C** ventral view of NJFU20180704005.

We collected 12 *Kurixalus* samples in April and July 2018 in north-western Zhejiang Province, People’s Republic of China (Fig. 1; 31.06°N, 119.85°E). Specimens in April were photographed and had buccal swabs taken for preliminary analysis and subsequently released (Fig. 2), with a follow-up collection expedition taking place in July, pending positive preliminary results for a potential novel species.

Specimens collected in July were humanely euthanised through cooling in line with Shine et al. (2015) and a subsequent application of 20% benzocaine applied to the venter (Torreilles et al. 2009). Specimens were deposited at the Biological Museum at Nanjing Forestry University (institutional code NJFU; Table 1). Genomic materials were collected from buccal swabs for the initial three individuals found in April (photographic vouchers deposited in the repository institution HerpMapper.org (institutional code HM) (HerpMapper 2020): HM 244044, HM 323117 and HM 323118) and thigh muscle tissues for the 11 specimens collected subsequently (Table 1). Genomic DNA was extracted from both swabs and tissues using a Qiagen DNA extraction kit (Blood and Tissue Kit; Qiagen, Germany) according to the manufacturer’s protocol.

**Table 1.** Samples and sequences used as taxa for the phylogenetic trees in this study.

Species	Sample voucher	GenBank accession number		Localities	Literature
		*12S-tRNA val-16S*	*TYR*		
*Kurixalusinexpectatus* sp. nov.	NJFU20180704001	MW115094	MW148393	Huzhou, Zhejiang, China	Present study
*Kurixalusinexpectatus* sp. nov.	NJFU20180704002	MW115093	MW148394	Huzhou, Zhejiang, China	Present study
*Kurixalusinexpectatus* sp. nov.	NJFU20180704003	MW115095	MW148395	Huzhou, Zhejiang, China	Present study
*Kurixalusinexpectatus* sp. nov.	NJFU20180704004	MW115092	MW148396	Huzhou, Zhejiang, China	Present study
*Kurixalusinexpectatus* sp. nov.	NJFU20180704005	MW115090	MW148397	Huzhou, Zhejiang, China	Present study
*Kurixalusinexpectatus* sp. nov.	NJFU20180704006	-	MW148398	Huzhou, Zhejiang, China	Present study
*Kurixalusinexpectatus* sp. nov.	NJFU20180705001	MW115088	MW148400	Huzhou, Zhejiang, China	Present study
*Kurixalusinexpectatus* sp. nov.	NJFU20180706001	MW115091	MW148401	Huzhou, Zhejiang, China	Present study
*Kurixalusinexpectatus* sp. nov.	NJFU20180706002	MW115096	MW148402	Huzhou, Zhejiang, China	Present study
*Kurixalusinexpectatus* sp. nov.	-	-	MW148399	Huzhou, Zhejiang, China	Present study
*Kurixalusinexpectatus* sp. nov.	NJFU20180706003	MW115089	MW148403	Huzhou, Zhejiang, China	Present study
*Kurixalusbaliogaster*	ROM29862	KX554476	KX554740	Krong Pa, Gia Lai, Vietnam	(Yu et al. 2017b)
*Kurixalusbaliogaster*	ROM29860	KX554475	KX554739	Krong Pa, Gia Lai, Vietnam	(Yu et al. 2017b)
*Kurixalusbaliogaster*	ROM33963	KX554474	KX554738	Krong Pa, Gia Lai, Vietnam	(Yu et al. 2017b)
*Kurixalusbanaensis*	ROM32986	GQ285667	GQ285799	Krong Pa, Gia Lai, Vietnam	(Li et al. 2009)
*Kurixalusbisacculus*	KUHE 19333	KX554473	KX554737	Phu Luanag, Loei, Thailand	(Yu et al. 2017b)
*Kurixalusbisacculus*	KUHE 19330	KX554472	KX554736	Phu Luanag, Loei, Thailand	(Yu et al. 2017b)
*Kurixalusbisacculus*	KUHE 35069	AB933291	KX554734	Pilok, Kanchanaburi, Thailand	(Yu et al. 2017b)
*Kurixalusbisacculus*	FMNH 261902	KX554471	KX554733	Kampot Dist, Prov, Cambodia	(Yu et al. 2017b)
*Kurixalusbisacculus*	FMNH 261901	KX554470	KX554732	Kampot Dist, Prov, Cambodia	(Yu et al. 2017b)
*Kurixalusbisacculus*	FMNH 261900	KX554469	KX554731	Kampot Dist, Prov, Cambodia	(Yu et al. 2017b)
*Kurixalusbisacculus*	FMNH 257903	KX554458	KX554699	Pakxong Dist, Champasak, Laos	(Yu et al. 2017b)
*Kurixalusbisacculus*	FMNH 256453	KX554456	KX554697	Nakai Dist, Khammouan, Laos	(Yu et al. 2017b)
*Kurixalusbisacculus*	FMNH 255656	KX554453	KX554694	Con Cuong Dist, Nghe An, Vietnam	(Yu et al. 2017b)
*Kurixalusbisacculus*	FMNH 255654	KX554451	KX554692	Con Cuong Dist, Nghe An, Vietnam	(Yu et al. 2017b)
*Kurixalusbisacculus*	FMNH 255661	KX554450	KX554691	VietnamTuong Duong Dist, Nghe An, Vietnam	(Yu et al. 2017b)
*Kurixalusbisacculus*	FMNH 255655	KX554452	KX554693	Con Cuong Dist, Nghe An, Vietnam	(Yu et al. 2017b)
*Kurixalusbisacculus*	FMNH 256452	KX554455	KX554696	Nakai Dist, Khammouan, Laos	(Yu et al. 2017b)
*Kurixalusbisacculus*	KUHE:19428	AB933290	KX554735	Nakon Sri Tamarat, Thailand	(Yu et al. 2017b)
*Kurixaluseiffingeri*	UMFS 5969	DQ283122	DQ282931	NanTou, Lu-Gu Chi-Tou, 900–1100 m, Taiwan	(Frost et al. 2006)
*Kurixaluseiffingeri*		AF458128			(Wilkinson et al. 2002)
*Kurixalusidiootocus*	UMFS 5702	DQ283054	DQ282905	NanTou, Tung Fu, 750 m, Taiwan	(Frost et al. 2006)
*Kurixalusidiootocus*		AF458129			(Frost et al. 2006)
*Kurixalusidiootocus*	SCUM 061107L	EU215547	EU215607	Lianhuachi, Taiwan	(Li et al. 2009)
*Kurixalusodontotarsus*	YGH 090132	GU227241	KX554683	Caiyanghe, Yunnan, China	(Yu et al. 2017b)
*Kurixalusodontotarsus*	YGH090130	GU227239	KX554681	Caiyanghe, Yunnan, China	(Yu et al. 2017b)
*Kurixalusodontotarsus*	Rao 14111401	KX554445	KX554680	Menglun, Yunnan, China	(Yu et al. 2017b)
*Kurixalusodontotarsus*	KIZ060821122	EF564456	KX554679	Menglun, Yunnan, China	(Yu et al. 2017b)
*Kurixalusodontotarsus*	YGH090177	GU227235	KX554677	Mengyang, Yunnan, China	(Yu et al. 2017b)
*Kurixalusodontotarsus*	YGH090176	GU227234	KX554676	Mengyang, Yunnan, China	(Yu et al. 2017b)
*Kurixalusodontotarsus*	YGH090175	GU227233	KX554675	Mengyang, Yunnan, China	(Yu et al. 2017b)
*Kurixalusodontotarsus*	Rao 14111307	KX554443	KX554674	Bada, Yunnan, China	(Yu et al. 2017b)
*Kurixalusodontotarsus*	Rao 14001643	KX554441	KX554672	Cangyuan, Yunnan, China	(Yu et al. 2017b)
*Kurixalusodontotarsus*	YGH090179	GU227236	KX554678	Mengyang, Yunnan, China	(Yu et al. 2017b)
*Kurixalusodontotarsus*	Rao 14102907	KX554442	KX554673	Cangyuan, Yunnan, China	(Yu et al. 2017b)
*Kurixalusverrucosus*	Rao 14102913	KX554440	KX554671	Yingjiang, Yunnan, China	(Yu et al. 2017b)
*Kurixalusverrucosus*	Rao 14102912	KX554439	KX554670	Yingjiang, Yunnan, China	(Yu et al. 2017b)
*Kurixalusverrucosus*	Rao 06308	KX554428	KX554657	Muotuo, Tibet, China	(Yu et al. 2017b)
*Kurixalusverrucosus*	Rao 06306	KX554427	KX554656	Muotuo, Tibet, China	(Yu et al. 2017b)
*Kurixalusverrucosus*	Rao 06302	KX554423	KX554654	Muotuo, Tibet, China	(Yu et al. 2017b)
*Kurixalusverrucosus*	Rao 06301	KX554422	KX554653	Muotuo, Tibet, China	(Yu et al. 2017b)
*Kurixalusverrucosus*	Rao 06201	KX554419	KX554651	Muotuo, Tibet, China	(Yu et al. 2017b)
*Kurixalusverrucosus*	Rao 06194	KX554416	KX554650	Muotuo, Tibet, China	(Yu et al. 2017b)
*Kurixalusverrucosus*	Rao 06193	KX554415	KX554649	Muotuo, Tibet, China	(Yu et al. 2017b)
*Kurixalusverrucosus*	CAS225128	GU227276	JQ060918	Nagmung, Kachin, Myanmar	(Yu et al. 2017b)
*Kurixalusverrucosus*	CAS 224381	GU227274	JQ060917	Nagmung, Kachin, Myanmar	(Yu et al. 2017b)
*Kurixalusverrucosus*	Rao 06202	KX554423	KX554654	Muotuo, Tibet, China	(Yu et al. 2017b)
*Kurixalusverrucosus*	Rao 06305	KX554426	KX554655	Muotuo, Tibet, China	(Yu et al. 2017b)
*Kurixalusverrucosus*	Rao 14102902	KX554430	KX554661	Muotuo, Tibet, China	(Yu et al. 2017b)
*Kurixalusverrucosus*	Rao 14102904	KX554432	KX554663	Nanjingli, Ruili, Yunnan, China	(Yu et al. 2017b)
*Kurixalusverrucosus*	Rao 14102905	KX554433	KX554433	Nanjingli, Ruili, Yunnan, China	(Yu et al. 2017b)
*Kurixalusverrucosus*	Rao 14102906	KX554434	KX554665	Nanjingli, Ruili, Yunnan, China	(Yu et al. 2017b)
*Kurixalusverrucosus*	Rao 14102910	KX554437	KX554668	Yingjiang, Yunnan, China	(Yu et al. 2017b)
*Kurixalusverrucosus*	Rao 14102909	KX554436	KX554667	Yingjiang, Yunnan, China	(Yu et al. 2017b)
*Kurixalus* sp.	MVZ Herp 223856	JQ060941	JQ060904	Tam Dao, Vinh Phu, Vietnam	(Yu et al. 2017b)
*Kurixalus* sp.	MVZ Herp 223863	JQ060943	JQ060921	Tam Dao, Vinh Phu, Vietnam	(Yu et al. 2017b)
*Kurixalus* sp.	MVZ Herp 223864	JQ060944	JQ060922	Tam Dao, Vinh Phu, Vietnam	(Yu et al. 2017b)
*Kurixalus* sp.	MVZ Herp 223865	JQ060945	JQ060923	Tam Dao, Vinh Phu, Vietnam	(Yu et al. 2017b)
*Kurixalus* sp.	MVZ Herp 223867	JQ060946	JQ060924	Tam Dao, Vinh Phu, Vietnam	(Yu et al. 2017b)
*Kurixalus* sp.	MVZ Herp 223868	JQ060947	JQ060925	Tam Dao, Vinh Phu, Vietnam	(Yu et al. 2017b)
*Kurixalushainanus*	HNNU A1180		EU215608	Mt. Diaoluo, Hainan, China	(Li et al. 2008)
*Orixaluscarinensis*	ROM39660	GQ285670	GQ285806	Sa Pa, Lao Cai, Vietnam	(Li et al. 2009)
*Romerusocellatus*	HN0806045	GQ285672	GQ285802	Mt. Wuzhi, Hainan, China	(Li et al. 2009)
*Romerusromeri*	KIZ 061205YP	EU215528	EU215589	Mt. Shiwan, Guangxi, China	(Li et al. 2009)
*Zhangixalusappendiculatus*	FMNH:267897				(Yu et al. 2017b)
*Zhangixalusappendiculatus*	FMNH 267896		JQ060926	Bukit Sarang, Sarawak, Malaysia	Yu et al. (2013)
*Zhangixalusnigropunctatus*	-	EU215533	EU924583	-	(Yu et al. 2017b)

### ﻿Molecular analyses

For all 11 individuals from which we extracted tissues, we amplified one mitochondrial and one nuclear gene fragment. For the mtDNA, we sequenced 827 bp from a section of the genes 12S rRNA, the complete tRNA-Valine (Val) and 16S rRNA, using the primer pair F0001 (5’-AGA TAC CCC ACT ATG CCT ACC C-3’), R1169 (5’-GTG GCT GCT TTT AGG CCC ACT-3’) (Wilkinson et al. 2002). For the nuclear gene, we sequenced 476 bp of the Tyrosine exon-1 (*TYR*), using the primer pair L2976 (5’-TGC TGG GCR TCT CTC CAR TCC CA-3’), H2977 (5’-AGG TCC TCY TRA GGA AGG AAT G-3’) (Bossuyt and Milinkovitch 2000).

The Polymerase Chain Reactions (PCR) were carried out in 20 µl reaction with 50 to 100 ng of template DNA, with 1.0 µl of each primer (10 mM). The final concentration of each PCR reaction resulted to 1.5 µl of MgCl_2_ (25 mM), 1.6 µl of dNTP (2.5 mM), 2.0 µl of 10× Buffer and 0.1 µl of TaKaRa Taq DNA polymerase (5 unit/µl). PCR amplifications were performed under the following thermal profiles: initial denaturation at 95 °C for 5 min, followed by 35 cycles with denaturation at 94 °C for 1 min, annealing at 55 °C for the mtDNA genes fragment and 54 °C for *TYR* for 1 min and extension at 72 °C for 1 min. The cycles were followed by a 10 min final extension at 72 °C. The amplified PCR products were sent for purification and sequencing to Cosmo Genetech Co. (Cosmo Genetech, Republic of Korea) on an ABI platform.

### ﻿Reconstruction of phylogenies and haplotype network

To reconstruct the independent and concantenated genes tree, we relied on two different datasets: (i) 827 bp-long fragments of mtDNA 12S rRNA, tRNA-Val and 16S rRNA (*n taxa* = 98), (ii) 486 bp-long fragments of sequences of protein-coding nuDNA Tyrosinase gene (*TYR*; n taxa = 110); and, (iii) 80 concatenated sequences of partial 12S rRNA (292 bp) and *TYR* (479 bp). We trimmed the sequences in each dataset manually and aligned the three sequences datasets indepedently using Clustal Omega (Sievers et al. 2011) in Geneious Prime (Kearse et al. 2012).

We calculated sequences similarity and estimated the genetic distance (or net evolutionary divergence) on the datasets of mtDNA 12S rRNA-trNA-Val-16S rRNA (*n* sequences = 98) and nuDNA *TYR* (*n* sequence = 110) using MEGA X (Kumar et al. 2018). We estimated the net average of evolutionary divergence between groups of sequences in each dataset; hence, we assigned 19 groups of species for 12S rRNA dataset and 16 groups of species for *TYR* dataset. In MEGA X, we conducted the analyses using the Maximum Compo-site Likelihood algorithm (Tamura et al. 2004) and modelled the rate variation amongst sites with a gamma distribution (shape parameter = 1). We considered differences in the composition bias amongst sequences in our evolutionary comparisons (Tamura and Kumar 2002), thus, all ambiguous positions were removed for each sequence pair using pairwise deletion option. These final datasets resulted in the totality of 116 positions, 301 sites for 12S rRNA and 110 positions, 486 sites for *TYR*.

For subsequent phylogenetic analyses, we downloaded supplemental sequences data of 98 homologous sequences of *Kurixalus* and *Zhangixalus* and other Rhacophoridae genera from Genbank (Wilkinson et al. 2002; Frost et al. 2006; Li et al. 2009; Nguyen et al. 2014; Yu et al. 2017a; Yu et al. 2018). GenBank accession numbers for both the new and previously deposited data are given in Table 1. We then created an initial alignment, based on nucleotide sequences with ClustalW2 (Larkin et al. 2007) and refined it manually. The final trimmed sequences resulted in 771 bp of concatenated 12S rRNA and *TYR* (*n* taxa = 79).

We used Partition Finder v. 2.1.1 (Lanfear et al. 2012) to determine the best-fit partitioning of the defined subsets. For the concatenated genes dataset, we defined four subsets by considering a fixed model for non-coding fragment and one subset for every single codon position with respect to the protein coding *TYR* gene fragments. Based on the Bayesian Information Criterion (BIC) values, we selected the following models for the following gene fragments: non-coding 12S rRNA (fixed subset): 1–292 (SYM+G) and protein coding *TYR* (subset 1): 293–771/1 (GTR+G); *TYR* (subset 2): 294–771/2 (K80+I+G) and *TYR* (subset 3): 295–771/3 (GTR+I+G). We used the models selected as a priori in further phylogeny analyses.

We built phylogenetic trees for all three datasets: mtDNA 12S rRNA-tRNA-Val-16S rRNA, protein coding nuDNA *TYR* and concatenated 12S rRNA-*TYR* using Bayesian Inference methodologies with MrBayes v.3.2.6 (Ronquist and Huelsenbeck 2003). For each tree dataset, we performed four separate analyses with 50 million generations of Markov Chain Monte Carlo and discarded the first 20 percent generations as burn-in until a convergence was reached (here we obtained > 0.005 split frequencies).

To test the presence of population differentiation using the *TYR* marker, we ran an analysis of molecular variance (AMOVA; Excoffier et al. 1992) using Arlequin v.3.5.2.2 (Excoffier and Lischer 2010). Here we used the AMOVA to test the three clades recovered from our phylogeny, based on *TYR* (Outgroup, Clade A and Clade B; see phylogenetic tree in Suppl. material 1: Fig. S2). In addition, we phased the diploid sequences of *TYR* gene (486 sites; *n* = 216) and analysed the haplotype using DnaSP v.5.0 (Librado and Rozas 2009). Before analysing the haplotypes, we assigned each haplotype group to its species, resulting 10 *Kurixalus* species and four closely-related species: *K.inexpectatus* sp. nov., *K.banaensis*, *K.baliogaster*, *K.bisacculus*, *Kurixalus* sp., *K.hainanus*, *K.odontotarsus*, *K.verrucosus*, *K.eiffingeri*, *K.idiootocus* and *Zhangixalusappendiculatus*, *R.ocellatus*, *R.romeri* and *Orixaluscarinensis*. Out of the 486 sites, we disregarded missing haplotypes and removed all invariable sites. Then, we converted the haplotype analysis in DnaSP v.5.0 to an RDF input file format and we built the reticulated haplotype network from the phased *TYR* in NETWORK v.10.2.0 (Fluxus Technology Ltd; UK) using the Median-joining method (Bandelt et al. 1995).

### ﻿Species delimitation and divergence time estimation

Relying solely on a distance-based method is insufficient. The coalescent-based species delimitation was determined as the most efficient method for comparative study of species delimitation in genus of *Kurixalus* (Yu et al. 2017a). To test the assumption that the individuals samples belonged to a new species rather than an exotic or invasive clade of *K.idiootocus*, we employed a topology testing and species delimitation approach using both the coalescent-based methods. First, we designed two competing topology species tree models: model 1 and model 2 with two independent datasets consisting respectively of 79 unlinked sequences of mtDNA 12S rRNA (292 bp) and nuDNA *TYR* (451 bp). Model 1 designated the new *Kurixalus* clade, *K.inexpectatus* sp. nov. as clumped within the clade of most closely related species, *Kurixalusidiootocus*, whereas Model 2 assigned *K.inexpectatus* sp. nov. as a new species, split from *K.idiootocus*. For a comparison between topologies, we ran a nested sampling analyses on species tree Model 1 and Model 2 with NS package implemented in BEAST v.2.6.6 (Bouckaert et al. 2019). We selected MCMC sub-chain length of 10,000 with particle count of 10 and an epsilon of 1.0×10^-9^ as parameters for each nested sampling analysis. Then, we evaluated topology of species trees of Model 1 and Model 2 by comparing the tree marginal L estimate (MLE) value and the Bayes factor obtained from the nested samplings. We calculated the Bayes factor with the following formula: Bayes factor = (MLE value of Model 1) – (MLE value of Model 2). We selected the best species tree model through the Bayes factor value, in which a positive Bayes factor is in favour of that particular model. We visualised the most likely species tree with Densitree (Bouckaert 2010).

Additionally, the recent study on the phylogeography of Taiwanese *Kurixalus* showed that the genus colonised the Island attributes through a land-bridge during the last glacial maxima (Yu et al. 2021). We further inferred the lineage origins and divergence between our focal taxa and *K.idiootocus* distributed in Taiwan Island. To do so, we estimated the time divergence of *Kurixalus* lineage by calibrating the species tree using an uncorrelated lognormal relaxed molecular clock with StarBeast RLC v.2.6.6 (Bouckaert et al. 2019). For both Model 1 and 2 datasets, we enforced three similar calibration points. Due to the absence of fossil records of Rhacophoridae in Asian mainland (Yu et al. 2021), we relied on paleogeological events for our primary calibration source. For secondary calibration, we adapted the range of molecular dating estimations of related literature (Pan et al. 2017; Yu et al. 2021). The three calibration points described as: (i) Emergence of *Zhangixalusnigropunctatus* in Southeast Asian and Chinese mainland ca. 11.39 Mya (High posterior density (HPD) 95%: 8.89–14.16; Pan et al. 2017), (ii) Emergence of stem group of South-eastern Asian clades of *Kurixalus* involves *K.verrucosus* group and its representative members ca. 7.4 Mya (HPD 95%: Yu et al. 2021) and (iii) Emergence of stem group of *K.eiffingeri* and *K.idiootocus* in Taiwan ca. 5.50 Mya (HPD 95%: 8.75 -3.25; Yu et al. 2021), simultaneously with the earliest island formation after physically separating from the south -astern mainland of China through the formation of the Taiwan strait (ca. 5.0-2.0 Mya modern shape; Teng 1990). We tested both birth-death and Yule priors on our species tree datasets and finally selected Yule as the best tree prior due to a better pattern of bifurcation for each crown node generated in trees. We ran four independent analyses, with MCMC chains of 20 million generations and 1,000 pre-burn-in steps for each dataset of model. We verified the convergence of the generated trees by evaluating the MCMC outputs with Tracer v.1.7.1 (Rambaut et al. 2018). Here, we ensured the values of effective sample size (ESS) for all parameters to be at least more than 1,000. We summarised a maximum clade credibility (MCC) tree for the calibrated tree time using TreeAnnotator, an application attached to BEAST v.2.6.6.

Finally, we projected the possible dispersal pathways, based on the molecular dating estimates focusing on the clade containing our focal species and Taiwanese *Kurixalus* on paleomaps using QGIS v.2.18.15. The oscillayers used to reconstruct the Plio-Pliocene maps was adapted from datasets provided in Gamisch (2019).

### ﻿Call data collection and extraction

The acoustic recordings of putative new *Kurixalus* species were recorded between April and July in 2018 at 24 °C with a linear PCM recorder (Tascam DR-40; California, USA) linked to a unidirectional microphone (Unidirectional electret condenser microphone HT-81, HTDZ; Xi’an, China). To determine the relationship with other species, we first compared the number of consecutive calls within a series of calls between the individuals recorded and *K.idiootocus*, *K.eiffingeri*, *K.berylliniris* and *K.wangi*. We then compared the call properties of the new population with that of *K.idiootocus* as it was the most closely-related species and the only species with the same number of calls within a series of consecutive calls (see results). The recordings of *K.idiootocus* were obtained in central Taiwan (23.9240 N, 120.8910 E) in July 2013, using a Tascam DR-70D digital recorder (TEAC Corporation, Tokyo, Japan) and a Sennheiser ME67/K6 directional microphone (Sennheiser Electronic GmbH & Co. KG, Hanover, Germany). All our recordings were recorded at a sampling rate of 44.1 kHz with 16-bit resolution. Temperature was recorded with a Tecpel DIT-517 infrared thermometer (between 22 and 25 °C; TECPEL Corporation, New Taipei, Taiwan). The genus emits a series of continuous notes, pooled in bouts of continuous calls. To compare *K.idiootocus* and the new *Kurixalus* population, we selected one entire series of consecutive calls for each individual and analysed 373 advertisement calls in total, including 238 calls for *K.idiootocus* (9 to 24 calls in a bout from each of 16 males) and 135 calls for the new population (9 to 21 calls in a bout for 9 males).

**Figure 3.**
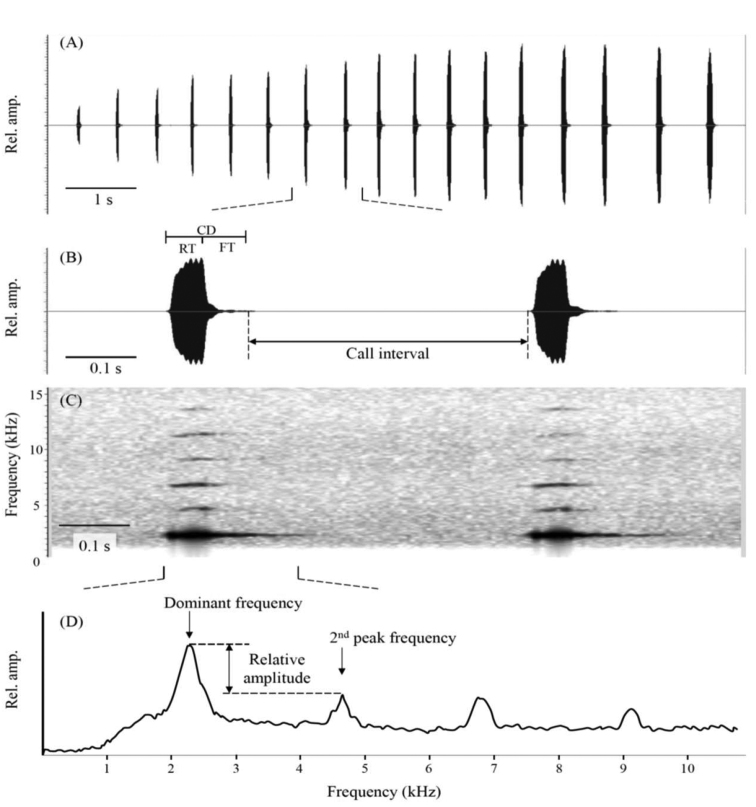
The call property measurements. This figure shows **A** the waveform of entire series of a consecutive call **B** the waveform **C** the spectrogram of two calls and **D** the spectral power distribution of a single call from the new *Kurixalus* population from Zhejiang, China. We extracted the number of calls in a bout, call interval, call duration (CD), rise time (RT), fall time (FT), dominant frequency (here also the max frequency), secondary peak frequency and the relative amplitude of two peaks.

We used Raven Pro v.1.5 (Cornell Lab of Ornithology 2011) to analyse our recordings. Nine properties, including six temporal and three spectral properties were measured in the two *Kurixalus* species (*K.idiootocus* vs. *K.inexpectatus* sp. nov): number of calls in a bout, bout length (s), call interval (ms), call length (ms), rise time (ms), fall time (ms), max frequency (kHz), 2^nd^ frequency (kHz), relative amplitude (dB). We measured the number of calls and the length of a series of consecutive calls. Call interval was measured as the duration from the end of a call to the beginning of the next call (Fig. 3A, B).

Call duration refers to the time between the onset and offset of a call. Rise time refers to the time between the onset of call and the local maximum in the waveform. Fall time is the time between the local maximum in the waveform and the offset of a call (Fig. 3B). Dominant frequency is the strongest frequency in the duration of a call and the secondary frequency is the maximum frequency of the second harmonic. Relative amplitude is the difference in amplitude between the dominant frequency and secondary high (dominant – secondary). In both species, the dominant frequency was the primary harmonic, as indicated through preliminary analyses and later confirmed by determining the fundamental frequency for a subset of calls as the reciprocal of the average period of the quasi-periodic fine-temporal waveform. All frequency measurements were based on 1024-point fast Fourier transformation and Hann windows and were made from the average power spectrum computed over the duration of a call.

### ﻿Call property analyses

We first corrected the calls for temperature variation by adjusting the value of each variable to the average temperature of all recordings using the equation originating from the linear regression of each focal variable in function of temperature. As the contribution of each call property for each individual is not independent of other call properties and consequently correlated, we used a principal component analysis (PCA) to convert those call properties into a set of values of linearly uncorrelated factors. The PCA provided four principal components with Eigenvalues larger than 0.5, explaining 95.7% of the total variance. We used a Discriminant Function Analysis (DFA) to classify the call properties and test for the correctness of group assignment. We then plotted the two significant PCs against each other to illustrate the divergence between the two species. Finally, to determine the differing call variables between the two clades, we used a Multivariate Analysis of Variance (MANOVA) to compare each call property between these two species.

### ﻿Morphometric measurements

We collected eighteen morphological measurements three times each and averaged the values for further analyses. Morphometric data were taken using digital calipers to the nearest 0.1 mm and included the following characters: snout-vent length (**SVL**), head width (**HDW**), distance between left and right articulations of jaw, head length (**HDL**), from the tip of the snout to the articulation of the jaw, snout length (**SNT**), from tip of snout to the anterior corner of the eye, horizontal eye diameter (**EYE**) from the anterior to the posterior corner of the eye, width of the upper eyelid (**UEW**), the horizontal length of the upper eyelid, internares distance (**IND**), the distance from nostril to eye (**DNE**), from the posterior border of nostril to anterior border of the eye, narrowest interorbital distance (**IOD**), greatest horizontal tympanum diameter (**TMP**), tympanum-eye distance (**TEY**) from anterior edge of tympanum to posterior corner of eye, hand length (**HND**) from distal end of radio-ulna to tip of finger III, radio-ulna length (**RAD**), forelimb length (**FLL**), distance from the proximate end of radio-ulna to distal end of finger III, thigh length (**THL**), distance from vent to distal end of femur, tibia length (**TIB**), foot length (**FL**) from proximal end of inner metatarsal tubercle to tip of toe IV and the length of the foot and tarsus (**TFL**), distance from tibio-tarsal joint to tip of toe IV. All specimens were measured by a single author (YY) to minimise sampling error. The dataset is available Suppl. materials.

To be able to compare with the morphometrics of other clades, we extracted data from the literature for all species available (Suppl. material 1: Table S4): *K.berylliniris*, *K.wangi*, *K.idiootocus*, *K.eiffingeri*, *K.bisacculus*, *K.lenquanensis*, *K.odontotarsus*. *K.yangi*, *K.naso*, *K.viridescens* and *K.ananjevae* (Kuramoto and Wang 1987; Matsui and Orlov 2004; Nguyen et al. 2014; Tao et al. 2014; Wu et al. 2016; Yu et al. 2017b; Yu et al. 2018; Zeng et al. 2021). The data were, however, incomplete for the variables TEY, TFL, THL, HND and RAD and these variables were removed from the analyses. We only kept data points that had a full dataset for the remaining 13 variables and were males to enable further morphological comparison without impact of sexual dimorphism. In total, we harvested data for 68 *Kurixalus* sp. individuals, including our samples. We then removed variation due to size difference between individuals by dividing each of the variables by the SVL of the matching individual. Furthermore and because of the low sample size for most species, we created two categories for the statistical analyses: one including our focal clade and the other one with all other non-focal species. The reasoning behind this segregation being that a clade would have to be extremely divergent to be morphologically different from all other species of the genera. We also created a subsection of the dataset including our focal clade (*n* = 12) and *K.idiootocus* as it is the most phylogenetically closely-related clade (*n* = 6).

### ﻿Morphometric analyses

As the variables were strongly correlated (Pearson’s correlation; Table 2), we decided to use a factor reduction statistical analysis to identify the independent dimensions of the morphological characters. The principal component analysis was set such that principal components were to be extracted if their Eigen value was > 1, under a varimax rotation. Variables were selected as loading into a PC if loading > 0.58 (Table 3). Once the PCs were extracted, we tested for significant differences between our focal clade and all other species through one-way ANOVA and then between our focal clade and *K.idiootocus* through a second one-way ANOVA. All analyses were run in SPSS (SPSS, Inc., Chicago, USA). Additionally, after standardising morphological measurements by SVL, we also ran a two-sample t-test comparing the putative new species (*n* = 12) and *K.idiootocus* (*n* = 8) on 12 of the morphological characters.

**Table 2.** Pearson correlation for all ten selected variables. We run a Pearson Correlation test (*n* = 68) to highlight the correlation between variables and highlight the need for a variable reduction analysis, such as a PCA. Cells in bold highlight significance.

		HDW	SNT	IND	IOD	UEW	EYE	TD	DNE	FLL	TFL	FL
HDL	r	0.84	0.34	0.23	0.44	0.60	-0.11	-0.13	0.09	0.86	0.45	0.08
	*p*	**< 0.001**	**0.005**	0.065	**< 0.001**	**< 0.001**	0.365	0.298	0.480	**< 0.001**	**< 0.001**	0.521
HDW	r		0.38	0.22	0.52	0.49	-0.06	-0.15	0.05	0.81	0.54	0.10
	*p*		**0.001**	0.072	**< 0.001**	**< 0.001**	0.634	0.218	0.698	**< 0.001**	**< 0.001**	0.432
SNT	r			-0.11	0.04	0.38	0.11	-0.25	-0.09	0.34	0.61	-0.05
	*p*			0.365	0.722	**0.002**	0.394	**0.044**	0.450	**0.005**	**< 0.001**	0.698
IND	r				0.01	0.48	-0.26	0.04	0.15	0.06	0.10	0.16
	*p*				0.961	**< 0.001**	**0.029**	0.773	0.230	0.644	0.439	0.195
IOD	r					0.07	0.11	-0.04	0.06	0.40	0.15	0.05
	*p*					0.565	0.383	0.731	0.648	**0.001**	0.209	0.695
UEW	r						-0.04	-0.16	0.09	0.46	0.48	0.06
	*p*						0.754	0.181	0.455	**< 0.001**	**< 0.001**	0.625
EYE	r							0.39	-0.70	-0.06	0.12	-0.42
	*p*							0.001	**< 0.001**	0.655	0.330	**< 0.001**
TMP	r								-0.47	-0.14	-0.23	-0.47
	*p*								**< 0.001**	0.273	0.059	**< 0.001**
DNE	r									-0.06	-0.17	0.73
	*p*									0.628	0.163	**< 0.001**
FLL	r										0.61	0.09
	*p*										**< 0.001**	0.471
TFL	r											0.13
	*p*											0.285

**Table 3.** Variables and results for the Principal Component Analysis and resulting ANOVA. Principal components were to be extracted if their eigenvalue > 1, under a varimax rotation. Variables were selected as loading into a PC if the value is > 0.58. In bold are variables retained as loading into one if the PCs. Based on the variables loading on to each of the PCs, we assigned PC1 to the general morphology and PC2 to the horizontal head structure. PC1 and PC2 were not significantly different between *Kurixalusinexpectatus* sp. nov. and other *Kurixalus* species under a one-way ANOVA, but they were significantly different between *Kurixalusinexpectatus* sp. nov. and *K.idiootocus*. The sample sizes used in the analysis were such as: *K.inexpectatus* sp. nov. *n* = 12, *K.idiootocus* n = 8; all *n* = 71; details in the Suppl. material S1).

	PC1	PC2
SVL	**0.90**	0.21
HDL	**0.92**	0.14
HDW	**0.96**	0.17
SNT	**0.84**	0.26
IND	**0.74**	-0.08
IOD	**0.85**	0.17
UEW	**0.84**	0.09
EYE	0.33	**0.84**
TMP	0.40	**0.59**
DNE	0.35	-**0.86**
FLL	**0.90**	0.21
TFL	**0.91**	0.25
FL	**0.85**	-0.28
Eigen value	8.25	1.92
Variance (%)	63.49	14.74
ANOVA all clades		
*χ2*	0.43	0.69
*F*	0.47	0.66
df1, df2	1,66	1,66
*p*	0.494	0.419
ANOVA focal clade-*K.idiootocus*		
*χ2*	1.42	1.39
*F*	13.35	14.56
df1, df2	1,66	1,66
*p*	**0.002**	**0.017**

## ﻿Results

### ﻿Sequence divergence, phylogenetic relationships and haplotype distribution

Our analyses resulted in minor differences in the evolutionary divergence between the 12S rRNA gene fragments of *K.inexpectatus* sp. nov. and *K.idiootocus* (mean = 0.0004 SD ± 0.0004). Similarly, the protein coding nuclear *TYR* between *K.inexpectatus* sp. nov. and *K.idiootocus* showed a comparatively smaller mean of substitution rate (mean = 0.0035 ± 0.0004; value marked with double asterisks (**) in Table 4) than that of other species groups (Table 4).

**Table 4.** Matrix of genetic distances between all pairs of sequences of protein-coding nuclear *TYR* between groups of sequences of 16 species rhacophorids species (*n* = 110). The 16 groups of species consisted of *Kurixalus* and *Rhacophorus* genera. Values in bold in the bottom left of diagonal matrix represent the means of estimate for each species divergence using maximum composite likelihood. Values of the upper right of diagonal matrix represents the standard deviation of each mean of divergence. The mean of distance between our proposed species *K.inexpectatus* sp. nov. and *K.idiootocus* noted with (**), which was higher to mean genetic distance of other pairwise species (values are in bold and marked with *).

Species	1	2	3	4	5	6	7	8	9	10	11	12	13	14	15	16
1 *K.ocellatus*		0.0071	0.0260	0.0287	0.0292	0.0290	0.0293	0.0292	0.0292	0.0302	0.0340	0.0286	0.0264	0.0206	0.0216	0.0275
2 *K.romeri*	**0.0144**		0.0254	0.0280	0.0290	0.0286	0.0290	0.0291	0.0290	0.0298	0.0338	0.0279	0.0258	0.0201	0.0218	0.0270
3 *K.inexpectatus*	**0.0780**	**0.0752**		0.0047	0.0057	0.0054	0.0056	0.0072	0.0057	0.0063	0.0104	0.0047	0.0025	0.0150	0.0147	0.0221
4 *K.banaensis*	**0.0862**	**0.0832**	**0.0081**		0.0058	0.0056	0.0057	0.0074	0.0059	0.0065	0.0104	0.0058	0.0043	0.0162	0.0147	0.0218
5 *K.baliogaster*	**0.0885**	**0.0876**	**0.0113**	**0.0109**		0.0006	0.0006	0.0035	0.0001	0.0028	0.0100	0.0063	0.0048	0.0171	0.0161	0.0229
6 *K.bisacculus*	**0.0877**	**0.0859**	**0.0108**	**0.0105**	**0.0007**		0.0001	0.0029	0.0004	0.0029	0.0102	0.0060	0.0044	0.0170	0.0157	0.0226
7 *Kurixalus* sp.	**0.0888**	**0.0875**	**0.0113**	**0.0110**	**0.0005**	-**0.0001**		0.0030	0.0001	0.0029	0.0102	0.0062	0.0046	0.0172	0.0160	0.0229
8 *K.hainanus*	**0.0884**	**0.0876**	**0.0161**	**0.0157**	**0.0046**	**0.0037**	**0.0036**		0.0035	0.0048	0.0114	0.0078	0.0064	0.0186	0.0167	0.0235
9 *K.odontotarsus*	**0.0887**	**0.0878**	**0.0114**	**0.0110**	0.0001*	0.0004*	0.0001*	**0.0046**		0.0029	0.0100	0.0063	0.0048	0.0171	0.0161	0.0229
10 *K.verrucosus*	**0.0926**	**0.0906**	**0.0134**	**0.0130**	**0.0035**	**0.0036**	**0.0037**	**0.0081**	**0.0035**		0.0110	0.0068	0.0054	0.0166	0.0162	0.0229
11 *Zhangixalusappendiculatus*	**0.1019**	**0.1009**	**0.0260**	**0.0256**	**0.0240**	**0.0250**	**0.0247**	**0.0290**	**0.0241**	**0.0278**		0.0109	0.0096	0.0224	0.0213	0.0283
12 *K.eiffingeri*	**0.0858**	**0.0829**	**0.0080**	**0.0109**	**0.0125**	**0.0120**	**0.0126**	**0.0173**	**0.0126**	**0.0143**	**0.0272**		0.0037	0.0157	0.0152	0.0227
13 *K.idiootocus*	**0.0797**	**0.0769**	0.0035^#*^	**0.0070**	**0.0086**	**0.0081**	**0.0086**	**0.0133**	**0.0086**	**0.0106**	**0.0231**	**0.0054**		0.0144	0.0138	0.0212
14 *K.carinensis*	**0.0605**	**0.0580**	**0.0421**	**0.0456**	**0.0494**	**0.0490**	**0.0497**	**0.0549**	**0.0496**	**0.0479**	**0.0666**	**0.0436**	**0.0401**		0.0111	0.0167
15 *R.nigropunctatus*	**0.0617**	**0.0613**	**0.0400**	**0.0397**	**0.0451**	**0.0438**	**0.0448**	**0.0469**	**0.0452**	**0.0457**	**0.0618**	**0.0412**	**0.0370**	**0.0274**		0.0145
16 *Rhacophorus* sp.	**0.0823**	**0.0794**	**0.0655**	**0.0632**	**0.0691**	**0.0678**	**0.0689**	**0.0711**	**0.0693**	**0.0693**	**0.0850**	**0.0668**	**0.0622**	**0.0460**	**0.0370**	

Overall, the Bayesian Inference (BI) trees inferred from both mtDNA 12S rRNA-tRNA-Val-16S rRNA and nuDNA *TYR* fragments showed strong patterns of genetic structures for the East Asian and Southeast Asian *Kurixalus* phylogeny relationship, recovering four strongly supported clades (Clades A, B, C and D; see the distributions of the clades and the phylogenetic tree in Suppl. material 1: Fig. S1), including two major clades (Clades A and B; Suppl. material 1: Fig. S2) within the *Kurixalus* lineage. Although showing a discordant topology for the clades distributed in Southeast Asia (Suppl. material 1: Figs S1 and S2), the mtDNA and nuDNA trees converged towards a similar phylogenetic position for *K.inexpectatus* sp. nov., highlighting a sister relationship with *K.idiootocus* (Suppl. material 1: Figs S1, S2).

**Figure 4.**
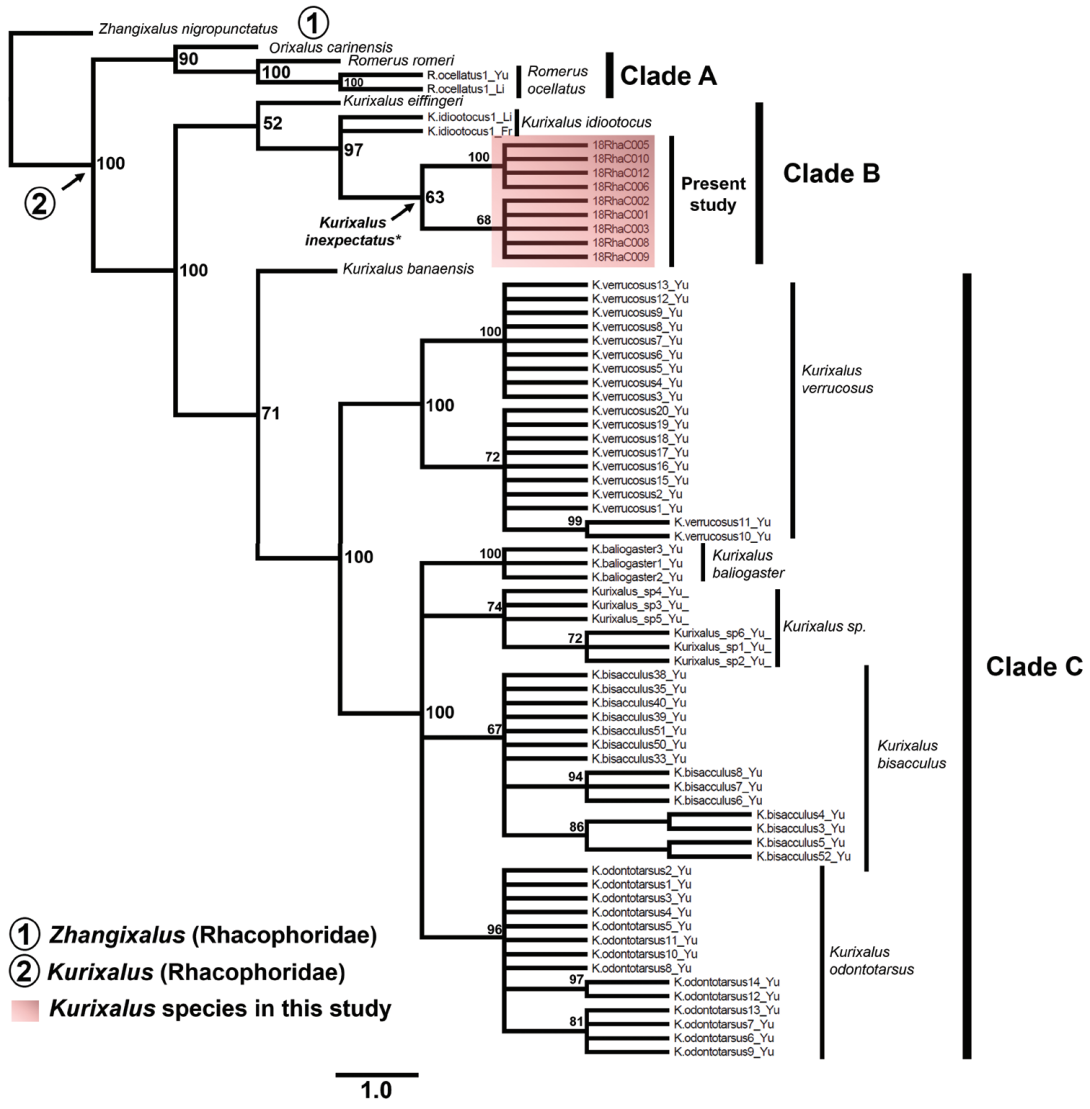
Bayesian Inference (BI) tree inferred from 79 sequences of concatenated 12S rRNA-*TYR* gene fragments. The three clades (Clades **A, B** and **C**) recovered in the phylogenetic tree are labelled accordingly. Clade **B** included the species *K.inexpectatus* sp. nov. described in the present study, indicated by the red box. The value of the node represents the Bayesian posterior probability (BPP) for each clade. The clades are marked with a solid bar and labelled in accordance with their specific name.

The phylogenetic relationship of concatenated gene fragments of partial 12S rRNA and *TYR* gene fragments recovered the three major clades within the *Kurixalus* genus with a Bayesian Posterior (BP) support of 90% for clade A, 52% for clade B, 71% for clade C (Fig. 4). Monophyletic clade A contained a Vietnamese *Kurixalus* (presumably as *K.carinensis*, but has been considered different from type species of *K.carinensis* in Myanmar) and *K.romeri* and *K.ocellatus* of Chinese mainland (BP = 100%; Fig. 4). Clade B recovered a monophyletic *Kurixalus* originated from Taiwan and south-eastern China. Here, the clades endemic to Taiwan, *K.eiffingeri* (Clade B, BP = 52%; Fig. 4) and *K.idiootocus* (BP = 97%; Fig. 4) were nested to the monophyletic clade of our *Kurixalus* sp. sampled in Zhejiang, south-eastern China (clade B, BP = 63%; Fig. 4). Clade B therefore supported a divergence between focal *Kurixalus* clade and *K.idiootocus*. Clade C comprised of a large nested monophyletic East Asian and Southeast Asian mainland *Kurixalus*, included clades of *K.banaensis* that were ranging in Vietnam and *K.verrucosus* (BP = 100%), *K.baliogaster* (BP = 100%), *Kurixalus* sp. (BP = 74%) *K.bisacculus* (BP = 67%) and *K.odontotarsus* (BP = 96%) originating from Western China, Tibet, Yunnan and the Eastern Indo-Chinese Peninsula: Vietnam, Thailand and Cambodia (Table 1; Fig. 4).

**Figure 5.**
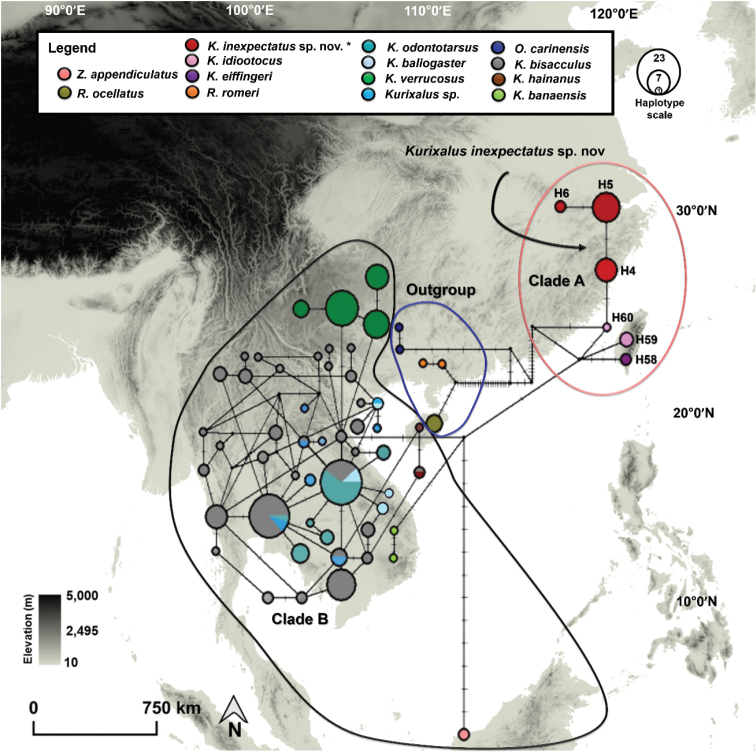
Haplotype network inferred from 216 phased nuDNA *TYR* sequences data (486 sites). The haplotype group for the focal Clade **A** comprised six representative haplotypes of *Kurixalus*. Clade **A** included three *K.inexpectatus* sp. nov. haplotypes. The size of each haplotype marker matches the haplotype scales. The colour coding matches with the name of the taxa in the legend. The colours used for the boundaries of Clade **A** and Clade **B** are coded similarly to the colours of their clades in the phylogenetic tree (Suppl. material 1: Fig. S2).

The AMOVA provided support to the genetic differentiation recorded while using the *TYR* marker as it identified 21.80% of variance within clades and 78.20% of variance between clades for the three main clades *Kurixalus* (*n* = 108; Fig. 4). The results of the AMOVA also provided a significant F_ST_ value (0.782; p < 0.05), showing that the three clades were significantly variable. The haplotype generated from the phased *TYR* fragment (*n* = 216), based in 477 trimmed sites, resulted in 62 haplotypes with a haplotype diversity (Hd) of 0.958 (Fig. 5). The distribution of haplotypes showed that six haplotype groups were representative of *Kurixalus* distributed in Taiwan Island and south-eastern China (Clade A; Fig. 5; see phylogenetic tree in Suppl. material 1: Fig. S2). Out of these six haplotype groups in Clade A, three of them represented *K.inexpectatus* sp. nov. (H4 – H6; Fig. 5). The origin of the haplotypes of *K.inexpectatus* sp. nov. corresponded to the haplotype groups of Taiwanese *Kurixalus*: *K.idiootocus* (H59- H60; Fig. 5) and *K.eiffingeri* (H 58; Fig. 5), whereas, Clade B contained a large portion of *Kurixalus* haplotype groups originating from south-eastern Asia, including *Z.appendiculatus* from Borneo and six *Kurixalus* species distributed across mainland Southeast Asia: Thailand, Laos, Cambodia and Vietnam (Fig. 5 and Suppl. material 1: Fig. S2). Clade B also included the representative haplotypes of *K.hainanus* distributed in Hainan Island and *K.verrucosus* haplotypes originating from Yunnan, south-western China.

### ﻿Species delimitation and divergence time estimates

**Figure 6.**
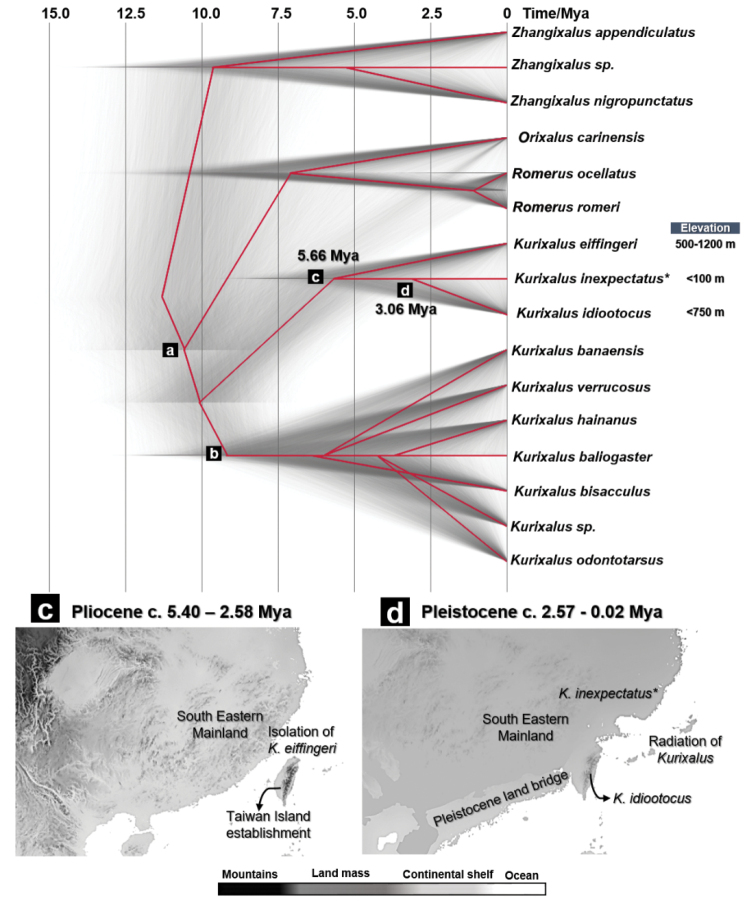
Calibrated species tree of Rhacophoridae represented by *Kurixalus*, *Orixalus*, *Romerus* and *Zhangixalus* distributed over East Asia and Southeast Asia. The species tree reconstructed from unlinked 12S rRNA and *TYR*. The asterisk (*) symbol indicates *Kurixalusinexpectatus* sp. nov. The highlighted lineages divergence noted with (**a–d**) and the time estimates are synchronised with datation in Table 6. Biogeography models **C** and **D** hypothesised early colonisation pathway of *Kurixalus* to Taiwan Island and potential glacial-driven refugia to the south-eastern mainland, projected on the Plio-Pleistocene oscillations models of.

The topology of the coalescent unlinked 12S rRNA and *TYR* tree supported the divergence of the focal *Kurixalus* clade from the most closely-related species, *K.idiootocus*. Nested sampling analyses on both species trees was favoured on the topology proposed by Model 2 (MLE = - 3688.252; Bayes factor: 651.011; Table 5). This topology provided support on the splitting between the lineages of *K.inexpectatus* sp. nov. distributed on south-eastern mainland and *K.idiootocus* distributed on Taiwan Island (Fig. 6), more so than a clumping between *K.inexpectatus* sp. nov. and *K.idiootocus* (see Model 2; Table 5).

**Table 5.** Nested sampling analysis results on two competing topology models for combined 12S rRNA and *TYR* using calibrated species trees. The values include summation of estimated Marginal L value with calculated Bayes factor for designated topology Model 1 and model 2. The positive value favoured the designated model. Topology Model 1 clumped *Kurixalusidiootocus* and *K.inexpectatus* sp. nov. as a single species. Topology Model 2 proposed *K.inexpectatus* sp. nov. as a new species and split from *K.idiootocus*. Bold values indicate the mean of nested sampling for each model.

Species tree topology	Nested sampling					Consensus
		Marginal likelihood estimate (MLE)	sqrt (H/N)	Standard deviation	Bayes factor (mean of MLE_1_-mean of MLE_2_)	
Model 1 (clumping)	1	-4339.441	6.099	6.008	-651.001	Model 1 is not favoured
	2	-4339.114	6.093	5.889		
	3	-4339.123	6.092	5.992		
	4	-4339.334	6.094	5.969		
	**Mean**	-**4339.253**	**6.095**	**5.965**		
Model 2 (splitting)	1	-3688.305	5.144	5.161	651.001	Model 2 is favoured
	2	-3688.131	5.143	5.458		
	3	-3688.361	5.145	5.279		
	4	-3688.211	5.143	4.925		
	**Mean**	-**3688.252**	**5.144**	**5.206**		

Our calibrated species tree of unlinked 12S rRNA and *TYR* gene fragments provided support on the earliest split between Asian lineages of *Kurixalus* and *Zhangixalus* to be dated in Mid-Miocene, ca. 11.17 Mya (Table 6; Fig. 5). This split-off was subsequently followed by the emergence of basal clade of *Kurixalus* in eastern Asia ca. 10.48 Mya [95% Highest Posterior Density (HPD): 8.16 – 12.98; node a; Table 6: Fig. 6]. Stem clade of *Kurixalus* distributed across the eastern Asian mainland, adjacent islands and south-eastern Asian mainland species group, consisting of members, such as *K.banaensis*, *K.verrucosus*. *K.baliogaster*, *K.odontotarsus*, *Kurixalus* sp. *K.hainanus* and *K.bisacculus* may have emerged ca. 9.14 Mya [6.87–11.12; node b; Table 6; Fig. 6]. Later, a stem clade of Taiwanese *Kurixalus* may have emerged, initiated by the isolation of *K.eiffingeri* in Taiwan and Ryukyu Islands ca. 5.66 Mya [3.32–8.07; node c; Table 6; Fig. 6]. Molecular dating estimates the lineage splitting between our proposed species, *Kurixalusinexpectatus* that distributed in south-eastern China and its sister clade, Taiwanese *K.idiootocus* to be in Late Pliocene to Pleistocene, ca. 3.06 Mya (5.82-0.01; node d; Table 6; Fig. 6).

**Table 6.** Molecular dating of 16 species of Asian rhacophorid frogs estimates the age of lineage separation between *K.inexpectatus* sp. nov. and *K.idiootocus*. The molecular dating estimation was using an uncorrelated lognormal relaxed clock with Yule prior on species tree inferred from unlinked 12S rRNA and *TYR* gene fragments of *Kurixalus* and rhacophorid taxa (*n* taxa = 79) distributed across Southeast Asia and East Asia.

Node	Clade (speciation event)	Node age (Mya)	
		Mean	HPD 95%
a	Emergence of stem clade of *Kurixalus* after split off from *Zhangixalus*	10.48	8.16–12.98
b	Stem clade of south-eastern and eastern Asian mainland group of *Kurixalus* (*K.verrucosus* + *K.baliogaster* + *K.odontotarsus* + *K.hainanus* + *K.bisacculus*)	9.14	6.87–11.50
c	Stem clade of Taiwanese *Kurixalus* group (isolation of *K.eiffingeri*)	5.66	3.32–8.07
d	Split off between Chinese mainland *K.inexpectatus* sp. nov. of south-eastern mainland and *K.idiootocus* of Taiwan Island	3.06	5.82–0.01

### ﻿Call properties

As the contribution of each call property for each individual is not independent of other call properties and consequently correlated, we used a principal component analysis (PCA) to convert those call properties into a set of values of linearly uncorrelated factors. We selected the principal components from the results of the PCA to cover as much as possible of the total variance, resulting in four PCs with Eigenvalues larger than 0.5 and explaining 95.7% of the total variance. We used a Discriminant Function Analysis (DFA) to classify the call properties and test for the correctness of group assignment. We then plotted the two significant PCs against each other to illustrate the divergence between the two species. Finally, to determine the differing call variables between the two clades, we used a Multivariate Analysis of Variance (MANOVA) to compare each call property between these two species.

Based on the descriptions of the advertisement call and the number of calls in a series of consecutive calls, we could first segregate the species into two groups matching with the phylogenetic clustering. We grouped the putative new *Kurixalus* species and *K.idiootocus* together, while *K.eiffingeri*, *K.berylliniris* and *K.wangi* were grouped together (Suppl. material 1: Tables S1, S2, S3). From here, when then compared the putative new *Kurixalus* species and *K.idiootocus*.

**Figure 7.**
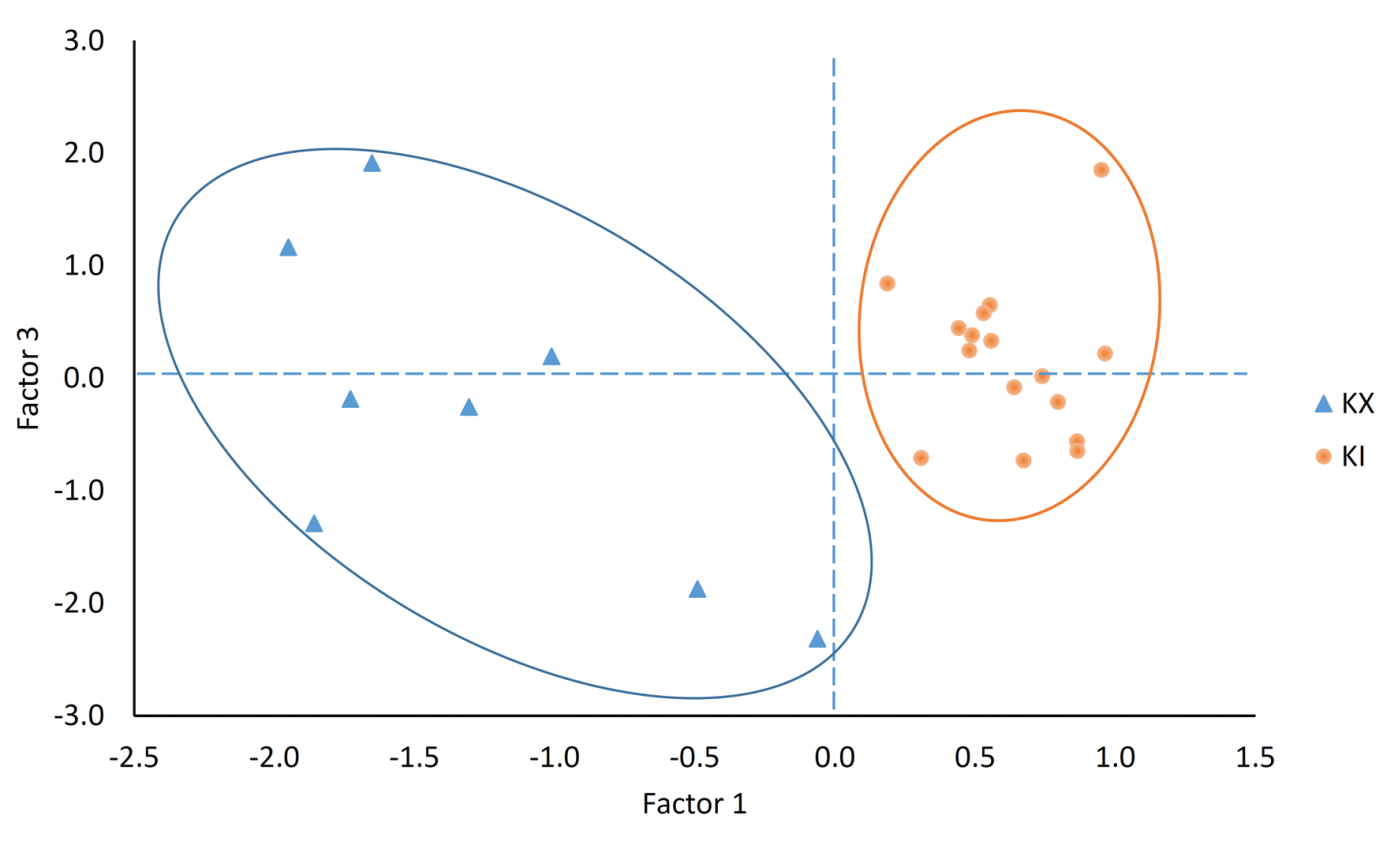
The PCA plot of the *Kurixalusinexpectatus* sp. nov. (KX) and *Kurixalusidiootocus* (KI).

The DFA on the four resulting PCs highlighted that only two PC1and PC3 were significantly different between the two species (PC1: Wilks’ Lambda = 0.93, F_(1,19)_ = 141.10, p < 0.001 ; PC3: Wilks’ Lambda = 0.17, F_(1,19)_ = 9.75, p = 0.005) and PC2 (Wilks’ Lambda = 0.11, F_(1,19)_ = 0.61, p = 0.442) and PC4 (Wilks’ Lambda = 0.12, F_(1,19)_ = 0.93, p = 0.345) were not. When plotting PC1 and PC3 against each other, a clear segregation of data was visible (Fig. 7). When comparing variables one by one for *K.idiootocus* and the new clade, the model was significant (MANOVA test; Wilks’ value = 0.087, F_9,14_ = 16.27, p < 0.001) and numerous variables were different from each other. In detail, the new clade had longer call intervals, longer call duration and dominant frequency (Table 7).

**Table 7.** The description results of advertisement call properties and the MANOVA test in *Kurixalusinexpectatus* sp. nov. and *Kurixalusidiootocus*. From the MANOVA test, the whole model Wilks’ value = 0.087, F_9,14_ = 16.27, p < 0.001. “*” indicate the data are not following the assumption of normal distribution (Shapiro-Wilk test, p < 0.05) and we transformed the data to their natural logarithm before doing statistical tests.

Call property	*K.inexpectatus* sp. nov. (*n* = 8)	*K.idiootocus* (*n* = 16)	F_1,22_	*p*
# of call in a bout	16.9 ± 3.9 (9–21)	14.9 ± 4.2 (9–24)	1.26	0.274
Bout length (s)	7.3 ± 2.9 (3.4–11.2)	3.6 ± 1.1 (1.9–5.5)	21.60	**< 0.001**
Call interval (ms)	376 ± 157 (115–539)*	211 ± 35 (159–278)	9.10	**0.006**
Call length (ms)	76.1 ± 11.1 (58–91)	34.8 ± 4.6 (28–43)	169.71	**< 0.001**
Rise time (ms)	38.1 ± 5.5 (29.5–46.0)	17.5 ± 2.4 (14–22)	167.48	**< 0.001**
Fall time (ms)	37.9 ± 5.5 (29.0–45.0)	17.5 ± 2.4 (14–22)	166.03	**< 0.001**
Max frequency (kHz)	2.30 ± 0.06 (2.20–2.39)	2.54 ± 0.08 (2.35–2.68)	54.20	**< 0.001**
2^nd^ frequency (kHz)	4.59 ± 0.11 (4.41–4.74)	5.05 ± 0.14 (4.71–5.33)	61.20	**< 0.001**
Relative amplitude (dB)	39.7 ± 8.3 (25.3–52)	35.5 ± 2.4 (30–39.7)	3.73	0.067

### ﻿Morphometrics

The unique PCA, used to identify the independent dimensions of the morphological characters between the individuals collected in this study and other *Kurixalus* sp. individuals, resulted in two PCs, with eigenvalues of 1.92 and 8.25, explaining a cumulated variation of 78.23% (Table 3). Based on the variables loading on to each of the PCs, we assigned PC1 to the general morphology and PC2 to the horizontal head structure (Table 3).

**Figure 8.**
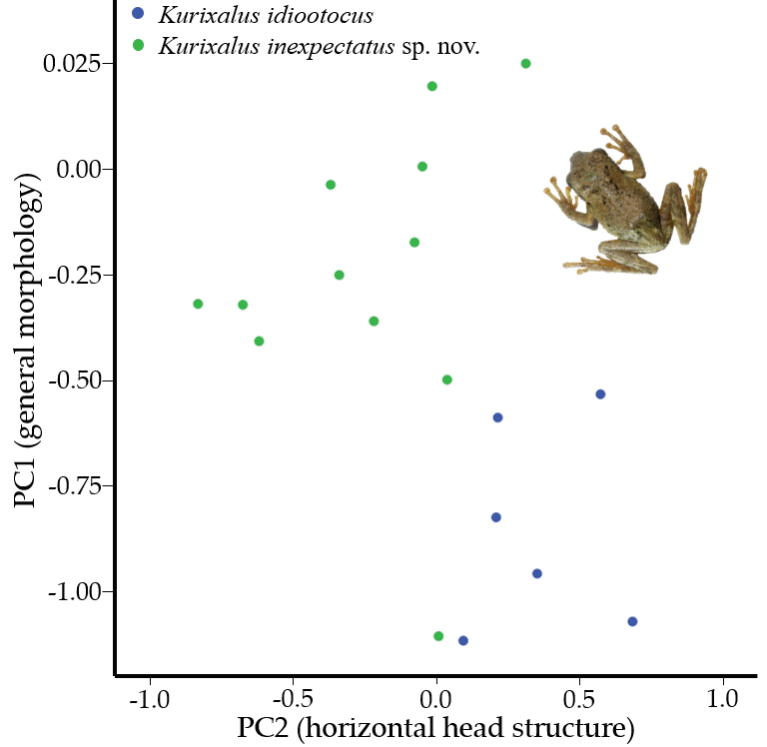
Plot of PC1 and PC2 resulting from the PCA and showing the non-clustering of morphological features between *Kurixalusinexpectatus* sp. nov. and *Kurixalusidiootocus*.

The results of the one-way ANOVA showed that there was no significant difference between the focal and non-focal groups for either of the PCs (Table 3). However, our focal clade and *K.idiootocus* were significantly different for both of the PCs (Table 3), such as PC1 (general morphology) *p* = 0.002 and PC2 (horizontal head structure) *p* = 0.017. When these variables were plotted against each other (Fig. 8), two non-clustering groups were visible, corresponding to variations between the measurements.

*K.inexpectatus* is morphologically most similar to *K.idiootocus*, its closest relative and, after standardising measurements by SVL, *K.inexpectatus* differs by having a relatively longer head length (34% vs. 33%), significantly shorter snout (13% vs. 15%; p < 0.001), significantly greater internasal distance (11% vs. 10%; p < 0.001), significantly smaller eye diameter (13% vs. 16%; p < 0.001), nearly significant wider tympanum diameter (7% vs. 6%; p = 0.06), significantly greater distance between the eyes and nares (8% vs. 7%, p = 0.03), significantly longer forelimb length (50% vs. 48%; p = 0.03), shorter tibia length (44% vs. 45%) and longer foot length (42% vs. 40%). Additionally, *K.inexpectatus* is further distinguished from *K.idiootocus* in having a tibio-tarsal articulation that extends beyond the anterior corner of the eye (versus centre of eye). *K.inexpectatus* can be differentiated from *K.bisacculus*, *K.hainanus*, *K.naso*, *K.odontotarsus*, *K.raoi*, *K.silvaenaias*, *K.verrucosus* and *K.yangi* by having an average adult SVL of less than 30 mm (27.5 – 31.8, × = 29.2) (vs. larger) (Yu et al. 2018; Hou et al. 2021; Zeng et al. 2021). *K.inexpectatus* can be further differentiated from *K.absconditus*, *K.baliogaster*, *K.banaensis*, *K.berylliniris*, *K.chaseni*, *K.eiffingeri*, *K.gracilloides*, *K.lenquanensis*, *K.motokawai*, *K.viridescens* and *K.wangi* by the presence of a pair of large, symmetrical dark blotches on the chest (vs. absent; Yu et al. 2018; Hou et al. 2021; Zeng et al. 2021). *Kurixalusinexpectatus* is distinguished from *K.ananjevae* by having limbs with serrated dermal fringes (vs. smooth; Yu et al. 2018; Hou et al. 2021; Zeng et al. 2021).

### ﻿Species description

#### ﻿ Kurixalus inexpectatus

Taxon classificationAnimaliaAnuraRhacophoridae

Messenger, Yang, Borzée, Chuang & Othman sp. nov.

530EDBF6-DA41-5DFD-87B3-32DFD6114DDC

http://zoobank.org/02D394DE-BB1C-4C17-BB70-656D68814C8F

##### Holotype.

**Figure 9.**
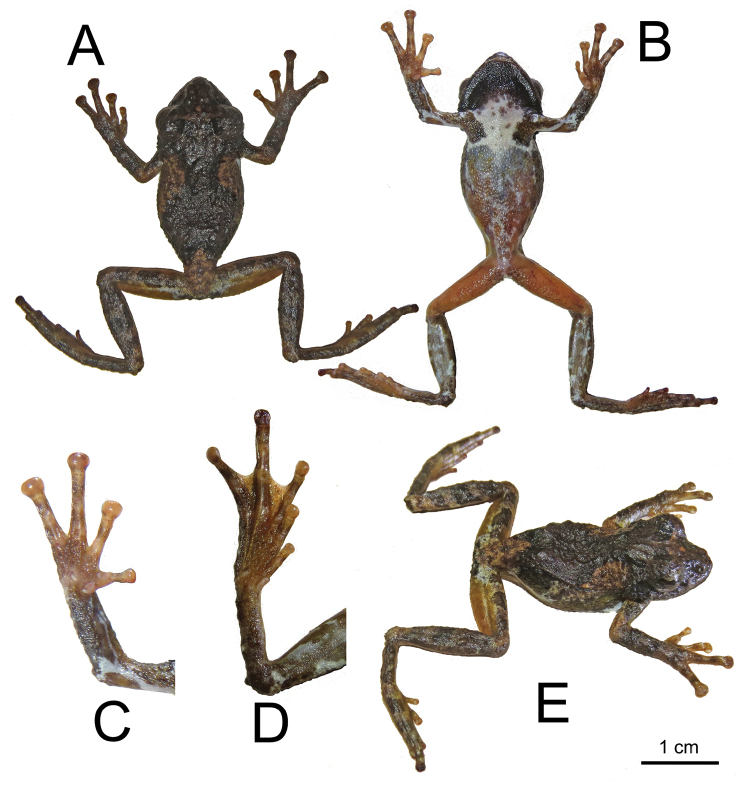
Holotype of *Kurixalusinexpectatus* sp. nov. **A** dorsal view **B** ventral view **C** right hand, ventral view **D** right foot, ventral view **E** dorsolateral view.

NJFU20180704001, an adult male (Fig. 9, Table 1), collected by Yi Yang (YY) on a dirt road in Chuanbu Village, north of Changxing 57 m a.s.l. on 4 July 2018 (Fig. 9).

##### Paratypes.

Five adult males, NJFU20180704002 – 20180704006, collected by YY on 4 July 2018 at the type locality. One adult male, NJFU20180705001, collected by YY on 5 July 2018 at the same location. Five adult males, NJFU20180706001-NJFU20180706005 collected by YY on 6 July 2018 at the same location.

##### Type locality.

Chuanbu Village (川步村), Changxing County, Huzhou City, Zhejiang Province, People’s Republic of China.

##### Etymology.

The epithet *inexpectatus* is Latin for “the unexpected.” This was chosen for several reasons. We selected this name because we had come to survey this region of China for different taxa. KRM came to this locale to survey for *Megophrys*. AB came to this locale to survey for *Dryophytes*. It was not only surprising to find this species while surveying for two other target genera, but upon realising the immense distance to the next closest population of *Kurixalus*, the discovery was even more unexpected. For an English and Chinese common name, we are recommending the name Changxing Treefrog (pronounced “Chang-shing” in English) 长兴原指树蛙 (cháng xīng yuán zhǐ shù wā).

##### Diagnosis.

The specimen matched the genus *Kurixalus*, based on the following characters: tips of digits enlarged to discs, with circum-marginal grooves; small-body size; pointed snout, forming a beak-like appearance; serrated dermal fringes along the outer edge of the forearm and leg; an inverted triangular-shaped dark brown mark between the eyes; dorsal “) (“ saddle-shaped marking; and a coarse dorsal and lateral surface with several small, irregular tubercles [7, 18, 29].

##### Comparisons.

*Kurixalusinexpectatus* sp. nov. is characterised and distinct from the majority of its congeners (19) by having a combination of being: (1) a small-sized species with an average adult size below 30 mm (in males); and (2) having two dark symmetrical pectoral blotches.

Genetically, the species is most closely related to *K.idiootocus* and is morphologically distinguished from this species by the combination of features: (1) having a tibio-tarsal articulation that extends beyond the anterior corner of the eye (versus the centre of eye); (2) having a significantly shorter snout relative to SVL; (3) a significantly greater internasal distance relative to SVL; (4) a significantly smaller eye diameter relative to SVL; (5) a nearly significantly wider tympanum diameter relative to SVL; (6) having a significantly greater distance between the eyes and the nares; (7) and by having a significantly longer forelimb length.

##### Description of holotype.

Adult male (SVL 29.4 mm); head width about the same as body, its length 37.9% of SVL; head slightly longer than wide in the holotype (11.1 mm vs. 11.0, respectively); snout pointed and slightly turned down, forming a small “beak-like” appearance typical in many rhacophorids; eye large, protuberant, ED 36.3% of HDL, 13.8% of SVL; pupil horizontal; tympanum distinct in form, but not distinct in texture or colour, its diameter 6.8% of SVL; nostrils protuberant; closer to the tip of the snout than the eye; vomerine teeth absent; tongue notched posteriorly; single internal vocal sac.

Relative length of fingers I < II < IV < III. Tips of all four fingers form discs with circum-marginal and transverse ventral grooves; relative width of discs is IV > III > II > I; nuptial pads absent; fingers lacking webbing at base; subarticular tubercles prominent and rounded; series of tubercles forming serrated dermal fringe along outer edge of forearm.

Heels overlapping when legs at right angle to body; relative length of toes is I < II < III < V < IV; toes moderately webbed at base; tips of toes expand to form discs with circum-marginal and transverse ventral grooves; toes discs are smaller than finger discs; relative size of toe discs I < V < IV < III < III; subarticular tubercles present, but not as obvious as hand.

Body is covered in numerous tubercles and dermal ridges. Ridges are present on the dorsum, but absent from the flanks and venter; tympanum also covered in tubercles.

##### Measurements of holotype (in mm).

The average of three measurements for each character is as follows: SVL 29.4, HDL 11.1, HDW 11.0, SNL 3.8, IND 3.3, IOD 3.3, UEW 3.1, ED 4.0, TD 2.0, TEY 0.9, DNE 2.7, FLL 15.7, THL 13.4, TL 14.5, FL 12.9, HND 9.1, RAD 6.7.

##### Colouration of holotype in life.

Light brown dorsum with white patch in the sacral region and extending a bit on to the femurs. Darker brown “) (“ dorsal saddle. Ventrally, white chest with brown colouration in the pectoral and axillary region. Ventral side of forelimbs have streaks of white and brown, almost like a marbled appearance. Ventral side of hind-limbs orange in the thigh and tibia region has the same brown and white marbled appearance present in the forelimbs. Palm of hand primarily light brown; sole of feet slightly darker than hand.

##### Colouration of holotype in preservation.

In preservation, the orange and light brown colours have faded, the darker brown has darkened compared to life. Pattern same as in life. Iris clouded. Chest white, throat black. Ventral side of arms black and white marbled appearance. Ventral side of tibia black and white marbled, similar to ventral aspect of forelimbs.

##### Variation.

As the holotype and paratypes of the new species are all male, sexual dimorphism cannot be ascertained. Aside from SVL, which is to be expected, the next characters which showed the greatest variation were FLL, TL, FL and TFL. Though the holotype has a head length longer than head width, most specimens had a head length shorter than head width. Colour varied between individuals, likely induced by temperature and/or time of day, as we observed this change first-hand. See Table 8 for variation amongst all specimens.

**Table 8.** Variation in morphological measurements amongst the holotype* and paratypes. Each character was measured three times, the values in the table represent the average of the three measurements. *Denotes holotype.

Specimen	SVL	HL	HW	SL	IND	IOD	UEW	ED	TD	TEY	DNE	FLL	THL	TL	FL	TFL	HND	RAD
20180704001*	29.4	11.1	11.0	3.8	3.3	3.3	3.1	4.0	2.0	0.9	2.7	15.7	13.4	14.5	12.9	19.9	9.1	6.7
20180704002	31.8	10.3	10.9	3.8	3.4	3.1	3.4	4.3	2.2	0.8	2.6	16.0	14.1	13.9	13.2	19.9	9.5	6.9
20180704003	29.7	10.2	10.1	4.0	3.3	3.0	3.1	3.9	2.1	1.1	2.7	14.7	13.1	12.8	12.5	18.7	8.6	7.0
20180704004	29.5	10.3	11.1	4.0	3.4	2.7	3.1	4.0	1.9	1.0	2.8	15.7	13.9	13.5	13.0	20.1	9.1	7.1
20180704005	29.4	9.6	10.7	3.4	3.1	3.0	2.8	4.0	1.8	1.0	2.5	15.2	13.5	13.6	12.7	19.6	8.6	6.8
20180704006	28.3	10.0	10.1	3.7	3.4	3.1	3.2	3.6	1.7	0.9	2.3	14.1	12.9	12.5	12.1	18.2	8.4	5.9
20180705001	28.6	9.7	10.7	4.2	3.3	3.1	2.9	3.2	1.8	1.2	2.6	14.2	13.3	12.9	10.9	17.6	8.0	6.4
20180706001	29.4	9.3	10.4	3.7	3.3	3.1	3.0	3.7	2.1	1.1	2.5	13.5	12.2	12.0	11.0	17.7	8.0	5.9
20180706002	29.8	10.1	10.9	3.5	3.4	3.4	3.0	4.0	2.0	0.8	2.3	15.1	13.9	13.4	12.9	19.4	8.8	6.3
20180706003	28.5	9.9	10.2	4.0	3.3	3.2	3.1	3.7	1.6	0.9	2.4	13.8	12.2	12.0	11.4	17.6	8.5	5.6
20180706004	29.0	9.9	10.4	3.7	3.4	3.2	2.9	3.8	1.9	0.9	2.5	14.5	12.9	12.9	12.0	18.6	8.7	6.3
20180706005	27.5	9.1	10.1	3.2	3.1	3.0	2.3	3.9	1.8	0.4	1.5	12.3	9.7	10.7	11.4	16.1	8.3	5.7

##### Description of eggs and tadpoles.

We did not find any eggs or tadpoles despite being present during the breeding season.

##### Distribution and ecology.

*Kurixalusinexpectatus* sp. nov. has been found calling as early as 26 April. Males would call from shrubs approximately 20 to 160 cm above temporary pools in and along roadside ditches. Temporary pools were 15 cm deep and up to 8 m long. In April, only sparse numbers of individuals were found calling. In July, full choruses could be heard, yet no individuals were found engaged in amplexus. No females have been found.

The vegetation primarily consisted of shrubs and secondary broad-leaved forest. No specimens were found in the adjacent bamboo forest.

##### Distribution.

Currently, the species is only known from the type locality, on the outskirts of the Wizard of Oz resort in Chuanbu Village, Changxing County, Huzhou City, Zhejiang Province, China. Surveys were made in the surrounding mountains for additional populations without success, including mountain ranges in Anhui and Jiangsu Provinces. The resort is situated at the southeast edge, in a northwest-to-southeast valley lower than 100 m in elevation. A creek comes from the hills, into a reservoir, which then flows about 2 km along the valley through the extent of the resort. The area was intended to be a plantation (unconfirmed, but suspected to be bamboo, based on the number of surrounding bamboo plantations), but in 2013, the land was set aside for the resort (pers. comm.). Now the resort consists of tea plantations, peach orchards, well-manicured grasses, a bamboo forest and miscellaneous shrubbery.

### ﻿Nomenclatural acts

The electronic edition of this article conforms to the requirements of the amended International Code of Zoological Nomenclature and, hence, the new names contained herein are available under that Code from the electronic edition of this article. This published work and the nomenclatural acts it contains have been registered in ZooBank, the online registration system for the ICZN. The ZooBank LSIDs (Life Science Identifiers) can be resolved and the associated information viewed through any standard web browser by appending the LSID to the prefix “http://zoobank.org/”. The LSID for this publication is: urn:lsid:zoobank.org:pub: 3CCB356B-F075-4EE5-8366-FE96B855F884. The new species name Kurixalusinexpectatus sp. nov. has been registered under LSID: urn:lsid:zoobank.org:act: 02D394DE-BB1C-4C17-BB70-656D68814C8F.

## ﻿Discussion

The molecular data and phylogeographic patterns presented here are supported by both call properties and morphological data, highlighting a significant segregation between *K.inexpectatus* and other species. The morphological analysis is robust in that *K.inexpectatus* is significantly different from closely-related clades in terms of calls and morphology and it has diverged from the most closely related species ca. 3.06 Mya.

In spite of the high genetic homogeneity between the 12S rRNA gene sequences of *K.inexpectatus* and its homologous species *K.idiootocus*, the haplotype distributions and phylogeny inferred from the nuDNA *TYR* gene fragment showed a distinction between the two clades. The incongruence in pattern of sequence divergences between 12S rRNA gene and *TYR* sequences may result from dissimilarities in the rate of evolution between mitochondrial and nuclear loci in the *Kurixalus* lineage. Accordingly, the phylogenetic tree inferred from nuDNA *TYR* gene and concatenating gene fragments of 12S rRNA and *TYR* also supported the sister species relationship between *Kurixalusinexpectatus* and *K.idiootocus* (subclade B2, BP = 97%) and recovered the monophyly of *K.inexpectatus* (Fig. 4, Suppl. material 1: Figs S1, S2).

Our haplotype network inferred from the nuDNA *TYR* gene sequences demonstrated the absence of identical haplotypes between *K.inexpectatus* and *K.idiootocus* (Clade A; Fig. 5), even if the three haplotype groups of *K.inexpectatus* shared the same ancestral haplotypes as *K.idiootocus* and *K.eiffingeri* (Clade A; Fig. 5). These results unconditionally rejected the possibility of *K.inexpectatus* to be an exotic or invasive population of *K.idiootocus* (Fig. 6). The pre-Pliocene estimates on the emergence of a stem group of Taiwanese *Kurixalus* (ca. 5.86 Mya [8. – 3.32]; Table 6; Fig. 5) matches with the establishment of the Island after the formation of the Taiwan Strait (Mio-Pliocene; ca. 5 Mya) (Teng 1990). The radiations of *Kurixalus* in Taiwan Island, dated to ca. 3.05 Mya, is highly consistent with the time estimates for the colonisation of Taiwan Island by *Kurixalus* (Pliocene, ca. 3.46 Mya)(Yu et al. 2021). Here our estimates regarding the segregation between *K.inexpectatus* and *K.idiootocus* is dated between the Plio-Pleistocene and the Holocene (biogeography models c and d; Fig. 6). This divergence time is supported by the formation of the Quaternary continental shelf which acted as temporary landmass connecting Taiwan Island to the mainland because of sea level fluctuations during glacial oscillations period. A similar phylogeography pattern is found in other anurans distributed in the south-eastern mainland of Asia and Taiwan Island, similarly sharing ancestry and having dispersed over the Pleistocene land-bridge (Yu et al. 2014; Othman et al. 2020). Here, the segregation between the two species is also supported by the difference in the elevational range of *K.idiootocus* (0 – 500 m) and *K.inexpectatus* (< 100 m; Fig. 6). Additionally, the recently described *K.silvaenaias* from Sichuan, China was discovered at 600 m elevation (Hou et al. 2021), a similar altitudinal preference as *K.idiootocus*.

Our results highlight the importance of advanced genetic analyses to support the conventional distance-based genetic divergence analysis and especially analyses on species delimitation (Table 5). Here, we suggested a splitting of lineage between *K.inexpectatus* and the Taiwanese endemic *K.idiootocus*, a similar taxonomic recommendation as that of Yu et al. (2017a), rejecting the synonymy between *K.hainanus* and *K.bisacculus* (Yu et al. 2010). Nonetheless, we recommend a comparative study including genomic, morphological and acoustic tools for *K.inexpectatus* and all other *Kurixalus* to resolve the taxonomy of the genus.

The lack of clear morphological characteristics is not unexpected for cryptic species and especially in treefrogs. However, identification based on range seems to be a reliable criterion. It is interesting that we did not find any individual in the bamboo forest while the genus is generally associated with this type of vegetation and further surveys may provide a different point of view. We recommend surveys on the contiguous mountain chain to determine the range of the species and the potential connectivity with other geographically related mountain ranges.

## ﻿Conclusion

Our work revealed a previous undescribed species of *Kurixalus* that was disjunct from the next closest population of the genus by nearly 700 km. The population was found in a highly developed region of northern China, yet surprisingly has gone unnoticed. This discovery reiterates the need to survey regions of the countryside that have been poorly studied. Such efforts should be especially considered in regions of high development, to ensure that potentially critically endangered species, previously unknown to science are not lost.

## Supplementary Material

XML Treatment for Kurixalus inexpectatus

## Supplementary materials

Supplementary material 1 Tables S1–S4, Figures S1, S2 Data type Docx file. Explanation note **Table S1.** The description of advertisement calls of five *Kurixalus* species. **Table S2.** The factor loading of principal analysis on call properties in *Kurixalusinexpectatus* sp. nov. and *Kurixalusidiootocus*. We listed the coefficients of correlation between call properties and their corresponding factors and listed out the Eigen values and accumulate explained variance of each Factor. **Table S3.** The factor scores of each individual for *Kurixalusinexpectatus* sp. nov. and *Kurixalusidiootocus* and the results of Discriminant Function Analysis (Wilks’ Lambda = 0.11, F (4.19) = 38.1, p < 0.001). This table shows the statistics and coefficient of determination of each factor and the square of Mahalanobis distance from each group centre, the post hoc probability (in brackets) to a group and the assigned group for each observation (individual). **Table S4.** Morphological data for *Kurixalusinexpectatus* sp. nov. used in this analysis. For the analyses, all measurements were adjusted for variations in body size, i.e. each value was divided by the SVL of the individual. The data presented here are not corrected for size. Data extracted from our samples and from the literature ananjevae (Kuramoto and Wang 1987; Matsui and Orlov 2004; Tao et al. 2014; Wu et al. 2016; Yu et al. 2017b; Yu et al. 2018; Zeng et al. 2021). **Figure S1.** Bayesian tree of Asian rhacophorids inferred from 98 sequences of 827 bp of partial mtDNA 12S rRNA-tRNA-Val-16S rRNA. Numbers above the lines or besides the nodes are given as Bayesian posterior probabilities (percentage). The species described in this study, *Kurixalusinexpectatus* sp. nov. are highlighted in the red-coloured box. **Figure S2.** Bayesian inference tree derived from 74 sequences of concatenated partial fragments of TYR. Numbers above the lines or besides the modes are given as Bayesian posterior probabilities (percentage). https://binary.pensoft.net/file/705228This dataset is made available under the Open Database License (http://opendatacommons.org/licenses/odbl/1.0/). The Open Database License (ODbL) is a license agreement intended to allow users to freely share, modify, and use this Dataset while maintaining this same freedom for others, provided that the original source and author(s) are credited.Kevin R. Messenger, Siti N. Othman, Ming-Feng Chuang, Yi Yang, Amaël Borzée
