# Supplementary material for: ﻿Description of a new Kurixalus species (Rhacophoridae, Anura) and a northwards range extension of the genus
Source: Zookeys. 2022 Jun 23;1108:15–49. doi: 10.3897/zookeys.1108.81725 (PMC9848858; doi:10.3897/zookeys.1108.81725)
Supplement: Supplementary material 1 — Tables S1–S4, Figures S1, S2 [file zookeys-1108-015_article-81725__-s001.docx]

Description of a new *Kurixalus* species (Rhacophoridae, Anura) and a northwards range extension of the genus

Kevin R. Messenger^1a,^ Siti N. Othman^2,^ Ming-Feng Chuang^3,^ Yi Yang^1,^ Amaël Borzée^2a^

^1^ Herpetology and Applied Conservation Lab, College of Biology and the Environment, Nanjing Forestry University, 159 Longpan Rd, Nanjing, Jiangsu 210037 China

^2^ Laboratory of Animal Behaviour and Conservation, College of Biology and the Environment, Nanjing Forestry University, 159 Longpan Rd, Nanjing, Jiangsu 210037 China

^3^ Department of Life Sciences and Research Center for Global Change Biology, National Chung Hsing University, No. 145 Xingda Rd., South Dist., Taichung 40227, Taiwan

^a^ Corresponding authors: [amaelborzee@gmail.com](mailto:amaelborzee@gmail.com), [kevinrmessenger@gmail.com](mailto:kevinrmessenger@gmail.com)

**SUPPLEMENTARY FILES**

## Supplementary tables

Table S1: The description of advertisement calls of five *Kurixalus* species.

| Species | N | # call in a bout | Call interval (s) | Call duration (ms) | Rise time (ms) | Fall time (ms) | Dominant freq (Hz) | 2nd peak freq (Hz) | Relative amplitude (1^st^ – 2^nd^ )(dB) | ref |
| --- | --- | --- | --- | --- | --- | --- | --- | --- | --- | --- |
| *Kurixalus* sp. nov. | 8 | 16.5 ± 3.7  (9 – 21) | 0.34 ± 0.16 (0.12 – 0.54) | 71.28 ± 17.88 (32.5 – 91.0) | 35.72 ± 8.71 (17 – 46) | 35.56 ± 8.63 (17 – 45) | 2306 ± 58 (2196 – 2390) | 4605 ± 108 (4407 – 4737) | 39.11 ± 8.03 (25.3 – 52.0) | Present study |
| *Kurixalus idiootocus* | 16 | 14.9 ± 4.2  (9 – 24) | 0.21 ± 0.03  (0.16 – 0.28) | 34.84 ± 4.56  (28.0 – 43.0) | 17.5 ± 2.35  (14.0 – 22.0) | 17.5 ± 2.37  (14.0 – 22.0) | 2538 ± 80  (2345 – 2678) | 5045 ± 143  (4708 – 5328) | 35.46 ± 2.43  (30.0 – 39.7) | Present study |
| *Kurixalus eiffingeri* | 36 | 1 | 1.75 ± 0.29 (1.10 – 2.40) | 38.04 ± 8.59 (24.0 – 55.0) | 17.33 ± 5.43 (10 – 33) | 20.32 ± 5.21 (12 – 32) | 2860 ± 125 (2670 – 3142) | 5693 ± 239 (5295 – 6182) | 36.64 ± 6.07 (24.8 – 45.9) | Present study |
| *Kurixalus berylliniris* | - | 1 | 3.20 ± 1.06 | 158 ± 56 | - | - | 2704 ± 35 | - | - | Wu et al. (2016) |
| *Kurixalus wangi* | - | 1 | 1.12 ± 0.23 | 99 ± 19 | - | - | 2841 ± 145 | - | - | Wu et al. (2016) |

Table S2: The factor loading of principal analysis on call properties in *Kurixalus inexpectatus* sp. nov. and *Kurixalus* *idiootocus*. We listed the coefficients of correlation between call properties and their corresponding factors and listed out the Eigen values and accumulate explained variance of each Factor.

|  | Factor 1 | Factor 2 | Factor 3 | Factor 4 | Factor 5 | Factor 6 | Factor 7 | Factor 8 | Factor 9 |
| --- | --- | --- | --- | --- | --- | --- | --- | --- | --- |
| # of call in a bout | -0.26 | 0.91 | 0.28 | -0.15 | 0.03 | -0.05 | 0.01 | < 0.01 | < 0.01 |
| Bout length | -0.88 | 0.24 | 0.33 | 0.22 | 0.04 | 0.12 | -0.02 | < 0.01 | < 0.01 |
| Call interval | -0.76 | -0.28 | 0.36 | 0.43 | 0.16 | -0.08 | 0.01 | < 0.01 | < 0.01 |
| Call length | -0.96 | 0.03 | -0.19 | 0.03 | -0.22 | -0.02 | < 0.01 | < 0.01 | -0.01 |
| Rise time | -0.96 | 0.04 | -0.19 | 0.04 | -0.22 | -0.01 | < 0.01 | -0.03 | < 0.01 |
| Fall time | -0.96 | 0.03 | -0.19 | 0.03 | -0.21 | -0.01 | < 0.01 | 0.02 | 0.01 |
| Max frequency | 0.91 | 0.05 | 0.20 | 0.20 | -0.30 | 0.02 | 0.06 | < 0.01 | < 0.01 |
| Secondary frequency | 0.91 | 0.06 | 0.23 | 0.18 | -0.28 | -0.03 | -0.06 | < 0.01 | < 0.01 |
| Relative amplitude | -0.54 | -0.42 | 0.57 | -0.43 | -0.14 | < 0.01 | < 0.01 | < 0.01 | < 0.01 |
| Eigen value | 6.10 | 1.15 | 0.85 | 0.51 | 0.35 | 0.03 | 0.01 | < 0.01 | < 0.01 |
| Accumulate explained variance (%) | 67.8 | 80.6 | 90.0 | 95.7 | 99.6 | 99.9 | 100.0 | 100.0 | 100.0 |

Table S3: The factor scores of each individual for *Kurixalus* *inexpectatus* sp. nov. and *Kurixalus idiootocus* and the results of Discriminant Function Analysis (Wilks’ Lambda = 0.11, F (4.19) = 38.1, *p* < 0.001). This table shows the statistics and coefficient of determination of each factor and the square of the Mahalanobis distance from each group centre, the post hoc probability (in brackets) to a group and the assigned group for each observation (individual).

| ID | Factor 1 | Factor 2 | Factor 3 | Factor 4 | KX (0.33) | KI (0.67) | Predict group |
| --- | --- | --- | --- | --- | --- | --- | --- |
| KX-01 | -0.49 | 1.02 | -1.87 | -2.21 | 8.1(1) | 37.0(< 0.01) | *Kurixalus* *inexpectatus* sp. nov. |
| KX-02 | -0.06 | 1.85 | -2.32 | 0.03 | 10.4(1) | 25.1(< 0.01) | *Kurixalus* *inexpectatus* sp. nov. |
| KX-03 | -1.73 | 0.69 | -0.18 | 0.94 | 2.7(1) | 48.4(0) | *Kurixalus* *inexpectatus* sp. nov. |
| KX-04 | -1.30 | -0.17 | -0.25 | 1.76 | 3.5(1) | 32.1(< 0.01) | *Kurixalus* *inexpectatus* sp. nov. |
| KX-05 | -1.95 | 0.66 | 1.17 | -0.25 | 3.8(1) | 48.3(0) | *Kurixalus* *inexpectatus* sp. nov. |
| KX-06 | -1.65 | -0.80 | 1.92 | -1.00 | 6.7(1) | 33.9(< 0.01) | *Kurixalus* *inexpectatus* sp. nov. |
| KX-07 | -1.86 | -0.27 | -1.29 | 0.46 | 6.0(1) | 64.6(0) | *Kurixalus* *inexpectatus* sp. nov. |
| KX-08 | -1.01 | -2.32 | 0.19 | -0.54 | 7.9(1) | 24.5(< 0.01) | *Kurixalus* *inexpectatus* sp. nov. |
| KI-01 | 0.48 | 0.83 | 0.25 | 2.14 | 37.6(0) | 4.9(1) | *Kurixalus idiootocus* |
| KI-02 | 0.49 | 0.43 | 0.38 | 0.20 | 30.2(0) | 0.4(1) | *Kurixalus idiootocus* |
| KI-03 | 0.56 | -0.32 | 0.33 | -0.05 | 32.7(0) | 0.1(1) | *Kurixalus idiootocus* |
| KI-04 | 0.55 | -0.19 | 0.65 | 0.00 | 35.3(0) | 0.3(1) | *Kurixalus idiootocus* |
| KI-05 | 0.19 | 1.04 | 0.84 | -2.02 | 24.4(<0.01) | 7.7(1) | *Kurixalus idiootocus* |
| KI-06 | 0.31 | -0.28 | -0.71 | -0.82 | 17.6(<0.01) | 4.2(1) | *Kurixalus idiootocus* |
| KI-07 | 0.64 | -0.54 | -0.08 | 1.30 | 37.6(0) | 1.8(1) | *Kurixalus idiootocus* |
| KI-08 | 0.97 | -1.00 | 0.22 | -0.79 | 46.8(0) | 2.5(1) | *Kurixalus idiootocus* |
| KI-09 | 0.86 | -0.44 | -0.56 | 0.07 | 36.2(0) | 0.7(1) | *Kurixalus idiootocus* |
| KI-10 | 0.74 | 0.11 | 0.02 | 0.32 | 36.0(0) | 0.2(1) | *Kurixalus idiootocus* |
| KI-11 | 0.53 | 0.79 | 0.58 | -0.29 | 31.8(0) | 1.0(1) | *Kurixalus idiootocus* |
| KI-12 | 0.87 | -1.02 | -0.65 | -0.15 | 37.1(0) | 1.7(1) | *Kurixalus idiootocus* |
| KI-13 | 0.67 | -1.30 | -0.73 | 0.00 | 31.9(0) | 2.4(1) | *Kurixalus idiootocus* |
| KI-14 | 0.44 | 0.19 | 0.45 | 0.79 | 31.7(0) | 0.7(1) | *Kurixalus idiootocus* |
| KI-15 | 0.80 | -0.87 | -0.21 | 0.12 | 38.2(0) | 0.9(1) | *Kurixalus idiootocus* |
| KI-16 | 0.95 | 1.91 | 1.85 | 0.02 | 63.1(0) | 9.4(1) | *Kurixalus idiootocus* |
| F (1,19) | 141.1 | 0.6 | 9.7 | 0.9 |  |  |  |
| p-value | < 0.001 | 0.443 | 0.006 | 0.346 |  |  |  |
| R square | 0.328 | 0.028 | 0.299 | 0.042 |  |  |  |

Table S4: Morphological data for *Kurixalus* *inexpectatus* sp. nov. used in this analysis. For the analyses, all measurements were adjusted for variations in body size, i.e. each value was divided by the SVL of the individual. The data presented here are not corrected for size. Data extracted from our samples and from the literature *ananjevae* (Kuramoto and Wang 1987; Matsui and Orlov 2004; Nguyen et al. 2014; Tao et al. 2014; Wu et al. 2016; Yu et al. 2018; Yu et al. 2017b; Zeng et al. 2021).

| Voucher | SVL | HL | HW | SL | IND | IOD | UEW | ED | TD | DNE | FLL | TL | FL |
| --- | --- | --- | --- | --- | --- | --- | --- | --- | --- | --- | --- | --- | --- |
| 18KurCX004 | 29.35 | 11.11 | 11.03 | 3.80 | 3.34 | 3.30 | 3.12 | 4.04 | 2.05 | 2.70 | 15.69 | 14.52 | 12.93 |
| 18KurCX005 | 31.77 | 10.29 | 10.92 | 3.75 | 3.44 | 3.14 | 3.36 | 4.26 | 2.23 | 2.62 | 16.01 | 13.93 | 13.22 |
| 18KurCX006 | 29.69 | 10.20 | 10.09 | 4.03 | 3.29 | 3.01 | 3.07 | 3.86 | 2.10 | 2.65 | 14.72 | 12.84 | 12.46 |
| 18KurCX007 | 29.48 | 10.31 | 11.08 | 3.97 | 3.40 | 2.69 | 3.05 | 3.97 | 1.86 | 2.76 | 15.74 | 13.50 | 13.04 |
| 18KurCX008 | 29.40 | 9.56 | 10.73 | 3.36 | 3.07 | 3.02 | 2.78 | 3.98 | 1.81 | 2.46 | 15.22 | 13.62 | 12.66 |
| 18KurCX009 | 28.26 | 10.04 | 10.10 | 3.66 | 3.38 | 3.10 | 3.18 | 3.63 | 1.66 | 2.25 | 14.10 | 12.49 | 12.11 |
| 18KurCX010 | 28.57 | 9.71 | 10.72 | 4.17 | 3.33 | 3.06 | 2.93 | 3.16 | 1.76 | 2.64 | 14.18 | 12.85 | 10.93 |
| 18KurCX011 | 29.38 | 9.35 | 10.41 | 3.72 | 3.27 | 3.08 | 2.98 | 3.71 | 2.15 | 2.49 | 13.48 | 12.00 | 10.99 |
| 18KurCX012 | 29.83 | 10.11 | 10.87 | 3.54 | 3.40 | 3.43 | 3.05 | 4.04 | 1.97 | 2.34 | 15.10 | 13.41 | 12.85 |
| 18KurCX013 | 28.47 | 9.91 | 10.16 | 3.99 | 3.26 | 3.17 | 3.07 | 3.66 | 1.58 | 2.39 | 13.84 | 11.99 | 11.38 |
| 18KurCX014 | 29.00 | 9.92 | 10.44 | 3.69 | 3.40 | 3.24 | 2.88 | 3.75 | 1.88 | 2.46 | 14.46 | 12.93 | 12.03 |
| 18KurCX015 | 27.50 | 9.13 | 10.12 | 3.19 | 3.13 | 2.96 | 2.32 | 3.88 | 1.81 | 1.47 | 12.33 | 10.65 | 11.36 |
| Kurixalus from ChangXing | 29.40 | 10.00 | 10.60 | 3.80 | 3.30 | 3.10 | 3.00 | 1.90 | 1.00 | 14.80 | 13.20 | 12.20 | 18.80 |
| Kurixalus idiootocus | 27.50 | 9.10 | 9.80 | 4.30 | 2.30 | 2.90 | 2.80 | 4.40 | 1.70 | 1.90 | 12.70 | 12.40 | 10.50 |
| Kurixalus bisacculus | 33.20 | 10.40 | 11.60 | 4.90 | 3.00 | 3.40 | 3.00 | 4.80 | 2.20 | 2.50 | 15.90 | 15.80 | 13.90 |
| Kurixalus lenquanensis | 27.00 | 8.50 | 9.50 | 3.70 | 2.70 | 2.90 | 2.40 | 4.10 | 1.60 | 1.80 | 13.20 | 12.40 | 12.00 |
| Kurixalus odontotarsus | 33.30 | 10.90 | 11.50 | 4.80 | 3.10 | 3.50 | 2.90 | 5.10 | 2.30 | 2.60 | 16.10 | 15.50 | 13.90 |
| Kurixalus yangi | 33.20 | 10.00 | 12.00 | 4.70 | 3.20 | 3.30 | 3.00 | 4.30 | 2.10 | 2.50 | 16.30 | 16.10 | 14.50 |
| Kurixalus naso | 31.30 | 10.50 | 11.40 | 5.00 | 3.40 | 3.10 | 3.30 | 4.30 | 1.70 | 2.40 | 16.10 | 15.90 | 14.10 |
| Kurixalus viridescens | 32.80 | 13.20 | 13.20 | 5.00 | 3.20 | 3.80 | 3.50 | 4.50 | 1.80 | 2.70 | 21.60 | 16.50 | 14.30 |
| Kurixalus naso | 31.60 | 10.30 | 11.30 | 5.00 | 3.30 | 3.20 | 3.40 | 4.30 | 1.60 | 2.10 | 15.90 | 16.00 | 13.90 |
| Kurixalus naso | 31.90 | 10.10 | 11.90 | 5.10 | 3.20 | 2.90 | 3.10 | 4.30 | 1.40 | 2.40 | 16.10 | 16.20 | 14.10 |
| Kurixalus naso | 32.50 | 11.10 | 12.00 | 5.30 | 3.70 | 3.20 | 3.70 | 4.70 | 2.10 | 2.50 | 17.70 | 16.50 | 15.40 |
| Kurixalus naso | 30.90 | 10.50 | 11.30 | 4.80 | 3.30 | 3.00 | 3.30 | 4.00 | 1.70 | 2.40 | 16.00 | 15.70 | 14.40 |
| Kurixalus naso | 31.40 | 10.50 | 11.30 | 5.10 | 3.40 | 3.10 | 3.30 | 4.30 | 1.80 | 2.30 | 16.10 | 16.20 | 13.90 |
| Kurixalus naso | 29.30 | 10.30 | 10.60 | 4.80 | 3.20 | 3.00 | 3.20 | 4.00 | 1.70 | 2.40 | 15.00 | 14.70 | 12.70 |
| Kurixalus viridescens | 28.70 | 11.70 | 11.20 | 4.70 | 3.10 | 3.30 | 3.20 | 4.00 | 1.40 | 2.70 | 17.90 | 15.50 | 12.40 |
| Kurixalus viridescens | 28.80 | 11.80 | 12.20 | 5.00 | 3.10 | 3.60 | 3.30 | 3.90 | 1.70 | 2.50 | 18.70 | 15.70 | 12.70 |
| Kurixalus viridescens | 30.90 | 12.60 | 12.60 | 5.00 | 2.80 | 3.40 | 3.50 | 4.80 | 2.10 | 2.80 | 20.50 | 15.10 | 13.70 |
| Kurixalus viridescens | 31.00 | 12.70 | 13.40 | 4.60 | 3.20 | 3.80 | 4.00 | 4.50 | 1.70 | 2.50 | 20.60 | 16.30 | 13.80 |
| Kurixalus viridescens | 32.80 | 12.90 | 13.00 | 4.90 | 3.30 | 3.70 | 3.60 | 4.60 | 1.90 | 2.50 | 22.50 | 17.00 | 13.70 |
| Kurixalus viridescens | 34.00 | 14.90 | 13.60 | 5.00 | 3.20 | 4.00 | 3.70 | 4.90 | 1.70 | 3.00 | 23.40 | 16.60 | 15.50 |
| Kurixalus viridescens | 34.10 | 14.30 | 14.10 | 5.30 | 3.50 | 3.80 | 3.30 | 4.20 | 2.10 | 2.70 | 22.80 | 16.50 | 15.70 |
| Kurixalus viridescens | 34.50 | 13.20 | 13.90 | 5.10 | 3.50 | 4.20 | 3.60 | 4.40 | 2.00 | 3.30 | 22.40 | 17.20 | 14.00 |
| Kurixalus viridescens | 36.30 | 13.40 | 13.80 | 5.20 | 3.30 | 4.30 | 3.30 | 4.20 | 1.70 | 2.20 | 24.10 | 18.10 | 16.20 |
| Kurixalus viridescens | 36.60 | 14.60 | 14.20 | 5.60 | 3.20 | 3.80 | 3.60 | 5.00 | 2.00 | 2.80 | 23.50 | 17.00 | 15.00 |
| Kurixalus lenquanensis | 27.00 | 8.50 | 9.50 | 3.70 | 2.70 | 2.90 | 2.40 | 4.10 | 1.60 | 1.80 | 12.30 | 12.40 | 12.00 |
| Kurixalus idiootocus | 27.50 | 9.10 | 9.80 | 4.30 | 2.60 | 2.90 | 2.80 | 4.40 | 1.70 | 1.90 | 12.70 | 12.40 | 10.50 |
| Kurixalus odontotarsus | 33.30 | 10.90 | 11.50 | 4.80 | 3.10 | 3.50 | 2.90 | 5.10 | 2.30 | 2.60 | 16.10 | 15.50 | 13.90 |
| Kurixalus bisacculus | 33.20 | 10.40 | 11.60 | 4.90 | 3.00 | 3.40 | 3.00 | 4.80 | 2.20 | 2.50 | 15.90 | 15.80 | 13.90 |
| Kurixalus yangi | 33.20 | 10.00 | 12.00 | 4.70 | 3.20 | 3.30 | 3.00 | 4.30 | 2.10 | 2.50 | 16.30 | 16.10 | 14.50 |
| Kurixalus naso | 31.30 | 10.50 | 11.40 | 5.00 | 3.40 | 3.10 | 3.30 | 4.30 | 1.70 | 2.40 | 16.10 | 15.90 | 14.10 |
| Kurixalus lenquanensis | 26.70 | 8.10 | 9.50 | 3.60 | 3.00 | 3.00 | 2.60 | 4.20 | 1.50 | 2.00 | 13.30 | 12.90 | 11.70 |
| Kurixalus lenquanensis | 26.10 | 8.20 | 9.10 | 3.60 | 2.60 | 2.90 | 2.60 | 4.00 | 1.40 | 1.90 | 12.80 | 12.20 | 11.60 |
| Kurixalus lenquanensis | 27.40 | 8.20 | 9.50 | 3.70 | 2.80 | 2.90 | 2.50 | 4.30 | 1.60 | 1.60 | 13.50 | 12.70 | 12.20 |
| Kurixalus lenquanensis | 27.10 | 8.30 | 9.50 | 3.90 | 2.60 | 2.90 | 2.50 | 4.10 | 1.40 | 2.00 | 13.10 | 13.00 | 12.00 |
| Kurixalus lenquanensis | 27.30 | 8.50 | 9.10 | 3.50 | 2.40 | 2.80 | 2.50 | 3.90 | 1.70 | 1.90 | 13.50 | 12.50 | 12.20 |
| Kurixalus lenquanensis | 27.20 | 8.10 | 9.10 | 3.70 | 2.50 | 2.80 | 2.30 | 4.10 | 1.60 | 1.70 | 13.80 | 12.60 | 12.50 |
| Kurixalus lenquanensis | 28.90 | 8.90 | 9.60 | 4.00 | 2.70 | 2.70 | 2.60 | 4.30 | 1.60 | 2.00 | 13.30 | 12.70 | 12.40 |
| Kurixalus lenquanensis | 27.10 | 8.20 | 9.10 | 3.70 | 2.70 | 2.80 | 2.50 | 4.30 | 1.80 | 1.60 | 13.70 | 11.80 | 12.70 |
| Kurixalus lenquanensis | 26.70 | 8.60 | 9.30 | 3.40 | 2.50 | 2.90 | 2.50 | 4.30 | 1.60 | 1.60 | 13.20 | 11.90 | 11.70 |
| Kurixalus lenquanensis | 27.10 | 8.90 | 10.00 | 3.80 | 2.80 | 3.10 | 2.00 | 4.00 | 1.60 | 2.00 | 13.10 | 12.20 | 11.90 |
| Kurixalus lenquanensis | 26.60 | 8.40 | 9.80 | 3.50 | 2.50 | 3.10 | 2.20 | 3.90 | 1.50 | 1.80 | 13.20 | 12.50 | 12.40 |
| Kurixalus lenquanensis | 26.90 | 8.70 | 9.70 | 3.80 | 2.80 | 3.00 | 2.50 | 4.30 | 1.50 | 1.60 | 13.10 | 12.50 | 12.00 |
| Kurixalus lenquanensis | 27.20 | 8.80 | 9.80 | 3.80 | 2.70 | 3.10 | 2.10 | 3.90 | 1.80 | 1.90 | 13.40 | 12.20 | 11.80 |
| Kurixalus lenquanensis | 25.00 | 8.70 | 9.70 | 3.70 | 2.60 | 2.90 | 2.30 | 3.90 | 1.50 | 1.80 | 12.40 | 11.60 | 11.20 |
| Kurixalus raoi | 28.20 | 8.50 | 10.40 | 4.10 | 2.60 | 3.20 | 2.40 | 3.90 | 1.50 | 2.40 | 13.90 | 12.90 | 12.20 |
| Kurixalus raoi | 31.10 | 9.50 | 10.90 | 4.50 | 2.60 | 3.30 | 2.40 | 4.00 | 1.80 | 2.80 | 14.50 | 13.80 | 12.60 |
| Kurixalus raoi | 30.20 | 9.20 | 10.60 | 4.40 | 2.60 | 3.10 | 2.50 | 4.20 | 1.70 | 2.40 | 14.20 | 13.60 | 12.50 |
| Kurixalus raoi | 30.80 | 9.40 | 10.80 | 4.50 | 2.70 | 3.00 | 2.50 | 3.90 | 1.70 | 2.60 | 14.30 | 13.70 | 12.60 |
| Kurixalus raoi | 29.60 | 9.20 | 10.20 | 4.20 | 2.70 | 3.10 | 2.60 | 3.80 | 1.60 | 2.50 | 14.00 | 13.40 | 12.20 |
| Kurixalus raoi | 32.20 | 9.80 | 11.70 | 4.80 | 2.90 | 3.40 | 2.80 | 4.10 | 1.80 | 2.80 | 16.00 | 15.50 | 14.20 |
| Kurixalus raoi | 29.60 | 9.30 | 10.40 | 4.10 | 2.30 | 2.80 | 2.60 | 4.20 | 1.70 | 2.30 | 14.10 | 13.50 | 12.80 |
| Kurixalus raoi | 38.60 | 11.90 | 13.30 | 5.50 | 3.70 | 4.20 | 3.30 | 4.70 | 2.00 | 3.10 | 19.00 | 17.60 | 16.70 |
| Kurixalus raoi | 30.80 | 9.40 | 10.20 | 4.40 | 2.60 | 3.40 | 2.50 | 3.90 | 1.60 | 2.50 | 14.60 | 13.70 | 12.70 |
| Kurixalus idiootocus | 27.50 | 9.20 | 9.70 | 3.90 | 2.80 | 3.00 | 2.80 | 4.30 | 1.60 | 2.00 | 13.10 | 13.10 | 11.30 |
| Kurixalus idiootocus | 29.30 | 9.50 | 10.20 | 4.30 | 2.70 | 3.00 | 3.10 | 4.40 | 1.90 | 2.10 | 13.40 | 12.70 | 11.90 |
| Kurixalus idiootocus | 26.60 | 9.30 | 9.80 | 3.90 | 2.60 | 2.90 | 2.90 | 4.30 | 1.70 | 1.90 | 13.10 | 12.20 | 11.30 |
| Kurixalus idiootocus | 25.70 | 8.80 | 9.50 | 3.80 | 2.70 | 2.90 | 2.80 | 4.20 | 1.60 | 2.10 | 12.60 | 11.90 | 10.90 |
| Kurixalus idiootocus | 37.50 | 12.00 | 12.90 | 5.50 | 3.90 | 4.10 | 3.40 | 5.30 | 1.90 | 3.00 | 18.20 | 17.30 | 15.30 |
| Kurixalus idiootocus | 28.90 | 9.30 | 10.00 | 4.10 | 3.00 | 3.30 | 2.70 | 4.40 | 1.60 | 2.20 | 13.80 | 12.40 | 11.40 |

## Supplementary Figures


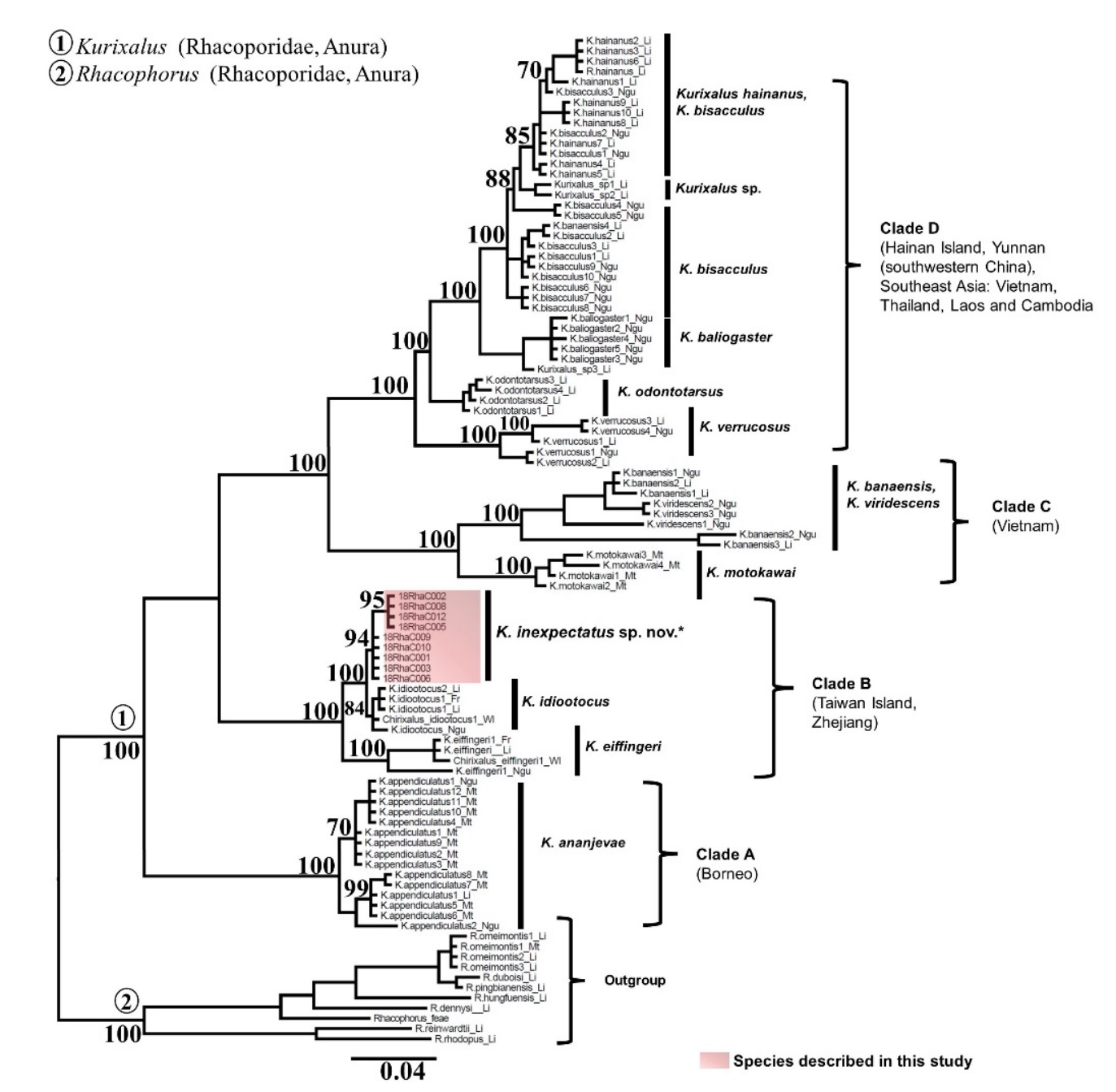


Figure S1: Bayesian tree of Asian rhacophorids inferred from 98 sequences of 827 bp of partial mtDNA 12S rRNA-tRNA-Val-16S rRNA. Numbers above the lines or besides the nodes are given as Bayesian posterior probabilities (percentage). The species described in this study, *Kurixalus inexpectatus* sp. nov are highlighted in the red-coloured box.


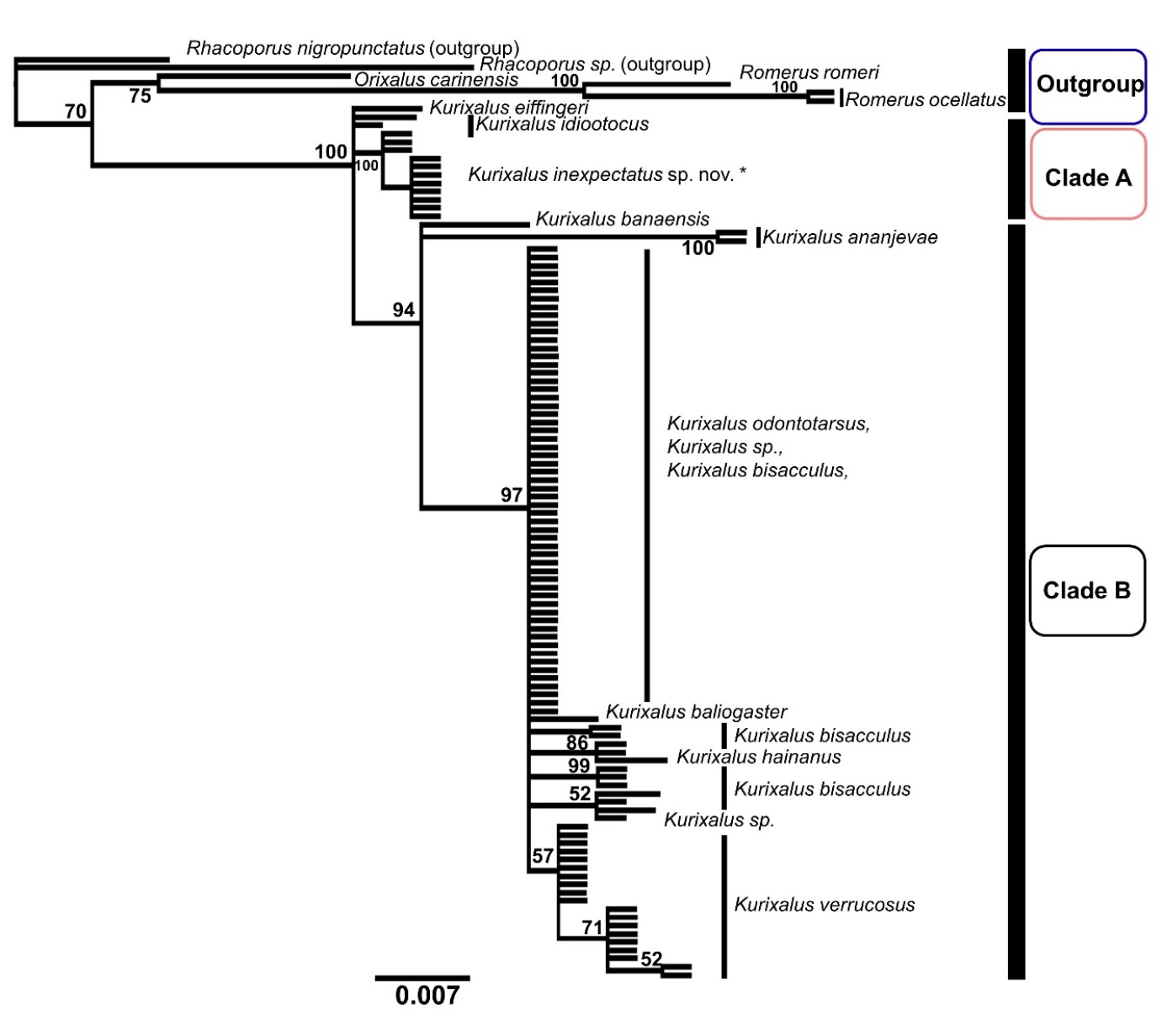


Figure S2: Bayesian Inference tree derived from 74 sequences of concatenated partial fragments of *TYR*. Numbers above the lines or besides the modes are given as Bayesian posterior probabilities (percentage).
